# Facial emotion processing and language during early-to-middle childhood development: An event related potential study

**DOI:** 10.1016/j.dcn.2021.101052

**Published:** 2021-12-17

**Authors:** Felicity J. Bigelow, Gillian M. Clark, Jarrad A.G. Lum, Peter G. Enticott

**Affiliations:** Cognitive Neuroscience Unit, School of Psychology, Deakin University, Geelong, Australia

**Keywords:** Facial emotion processing, Language, Event-related potentials, Early-to-middle childhood

## Abstract

Facial emotion processing (FEP) is critical to social cognitive ability. Developmentally, FEP rapidly improves in early childhood and continues to be fine-tuned throughout middle childhood and into adolescence. Previous research has suggested that language plays a role in the development of social cognitive skills, including non-verbal emotion recognition tasks. Here we investigated whether language is associated with specific neurophysiological indicators of FEP. One hundred and fourteen children (4–12 years) completed a language assessment and a FEP task including stimuli depicting anger, happiness, fear, and neutrality. EEG was used to record key event related potentials (ERPs; P100, N170, LPP at occipital and parietal sites separately) previously shown to be sensitive to faces and facial emotion. While there were no main effects of language, the P100 latency to negative expressions appeared to increase with language, while LPP amplitude increased with language for negative and neutral expressions. These findings suggest that language is linked to some early physiological indicators of FEP, but this is dependent on the facial expression. Future studies should explore the role of language in later stages of neural processing, with a focus on processes localised to ventromedial prefrontal regions.

## Highlights

• We examine the relationship between language and neurophysiological measures of FEP ERPs across development.
• FEP ERPs of interest included P100, N170 and LPP.
• Findings suggest relationship between language and neurophysiological measures may differ to behavioural measures of FEP.
• Evidence for language playing an interactive role across early and later ERPs.
• The interactive role of language appears to be emotion specific.

## 1 Introduction

Facial emotion processing (FEP) is a key component of social cognition, providing children with crucial information about their social environment. A happy face may suggest a positive social interaction with limited threat, whilst an angry or fearful face may suggest the presence of a threat within the social interaction or the surrounding environment. Indeed, infants as young as 5–7 months preferentially allocate attention to fearful over happy faces (Kotsoni et al., 2001; Leppänen et al., 2007; Peltola et al., 2009; Xie et al., 2019). This suggests that, at a rudimentary level, infants differentiate between faces displaying stereotyped scared versus happy expressions. Developmentally, the ability to process facial expressions improves in early childhood and continues to be fine-tuned throughout middle childhood and into adolescence (Batty and Taylor, 2006, Herba et al., 2006, Meaux et al., 2014). This suggests that the neural systems underlying the processing and differentiation of facial emotions are forming during early childhood (Conte et al., 2020, De Haan et al., 2002; Grossmann and Johnson, 2007, Jessen and Grossmann, 2016). Given this development is occurring in parallel with other social cognitive skills such as language, it is important to consider how these skills might influence one another during childhood.

### 1.1 Relationship between FEP and language

Language has been suggested to play a significant role in children’s FEP ability (Ruba and Repacholi, 2020). Previous research has demonstrated a relationship between behavioural measures of FEP and language in children across both early (Strand et al., 2016) and middle childhood (Beck et al., 2012, Pons et al., 2003; though see Herba et al., 2006 for contradictory findings). That is, language ability was found to be positively associated with behavioural performance on facial emotion recognition tasks requiring the verbalisation of an emotion category. Furthermore, these findings have been replicated across early and middle childhood using largely non-verbal affect recognition tasks (Bahn et al., 2021, Bigelow et al., 2021). This suggests that the role language has on FEP may extend beyond verbalising emotion categories, influencing the accuracy with which facial expressions are perceived. It is possible that language may assist in the integration of social cognitive skills necessary when processing a facial emotion.

Theoretically, the psychological constructionist perspective argues that the development of emotion concept knowledge, such as the labelling of emotion categories, are dependent upon language (Brooks and Freeman, 2018). This perspective posits that the acquisition of emotion categories is gradual. Thus, accuracy on behavioural emotion recognition tasks would be expected to gradually improve during development alongside the formation of increasingly nuanced emotion categories and social cognitive skills. It is theorised that the labelling of an emotion (e.g., “angry,” “happy”) enables the construction of a discrete emotional category (Fugate et al., 2018, Lindquist and Gendron, 2013). In turn, this emotion word assists in the transformation of often ambiguous affective states within a given context, into discernible categories of emotion (e.g., anger) (Brooks et al., 2017, Nook et al., 2015). Given the rapid development of language skills during childhood, there appears to be a strong theoretical argument for the association between language and the processing of facial emotions. Previous research has found that emotion categories are initially formed using a positive or negative dichotomy, before discrete emotion categories emerging in early childhood become increasingly nuanced alongside development (Nook et al., 2017, Widen and Russell, 2003). Furthermore, previous research has shown a positive association between language ability in early childhood and the labelling of emotion categories in middle childhood (Bigelow et al., 2021, Griffiths et al., 2020, Kårstad et al., 2015). It is therefore important to consider the potential developmental influence of the association between language and FEP. However, it is currently unknown whether these behavioural findings are replicated at a neurophysiological level, and how language may relate to these neurocognitive processes of FEP across development.

### 1.2 Face-sensitive event related potentials

The neural processes that support FEP can be examined using electroencephalography (EEG). In this research a common approach used is to present a facial expression and measure the evoked level of activity (amplitude) and speed (latency) via event related potentials (ERPs) (e.g., (Batty and Taylor, 2006; de Haan et al., 1998; Meaux et al., 2014). This paradigm may provide insight into which aspect/s of FEP language relates to, thus allowing us to examine the relationship between neural processes that support FEP and language. Several ERPs have been shown to be sensitive to faces, with research additionally suggesting developmental changes (Batty and Taylor, 2006, Meaux et al., 2014; Miki et al., 2011) and emotional sensitivity across childhood (Leppänen et al., 2007). The P100 is a positive peak, maximal at occipital sites, occurring roughly 100 ms after stimulus onset, thought to reflect the low-level processing of visual stimuli (Herrmann et al., 2005, MacNamara et al., 2016). Primarily, studies have found no emotion effects on the P100 (O’Connor et al., 2005, Usler et al., 2020), however, this is contested by some studies (D’Hondt et al., 2017). Previous FEP studies investigating P100 in children have generally reported a decrease in P100 amplitude (Batty et al., 2011; Meaux et al., 2014) and a reduction in P100 latency (Batty et al., 2011; Batty and Taylor, 2006) with increasing age. The N170 is a negative deflection, occurring roughly 170 ms post stimulus, and is maximal at posterior sites (Bentin et al., 1996). With respect to FEP, the N170 is typically associated with the initial processing of face characteristics (Eimer and Holmes, 2007). Previous studies have primarily found no emotion effect on the N170 in children (Apicella et al., 2013, Usler et al., 2020), however some studies have reported emotion effects (Battaglia et al., 2017, Batty and Taylor, 2006). Previous FEP studies support a non-linear relationship between age and N170 amplitude in children, however, overall, N170 amplitude tended to become stronger with increasing age (though for contrasting results see (Battaglia et al., 2007). Primarily, N170 latency has been reported to decrease with increasing age (Batty and Taylor, 2006, Meaux et al., 2014). Given the P100 and N170 reflect the initial stages of FEP, the development of these components during childhood are unlikely to be emotion specific.

The Late Positive Potential (LPP) is an emotion-sensitive, slow positive deflection beginning at roughly 400 ms post stimulus onset and often measured at occipital and parietal sites in children (Dennis and Hajcak, 2009). Several studies (Chronaki et al., 2018, Kujawa et al., 2012) have reported differences across LPP activity recorded at occipital and parietal sites in early-to-middle childhood populations. Therefore, to explore developmental patterns, the LPP is often measured separately at occipital and parietal sites in children. The LPP is typically associated with the processing of elaborate facial emotions (Hua et al., 2014, Schupp et al., 2004). The LPP has repeatedly shown an effect of emotion, with larger amplitudes recorded for expressive faces when compared to neutral faces (Keil et al., 2018, Kujawa et al., 2012, Usler et al., 2020). Furthermore, some evidence suggests that emotion effects may vary with facial expressions (Keil et al., 2018). For example, Gu et al. (2019) found that fearful faces elicited stronger LPP when compared to happy or neutral expressions across participants in middle childhood. However, other studies (see MacNamara et al., 2016) have reported no expressive facial emotion specific effects. Despite minimal and mixed literature, previous research investigating LPP amplitude and age has illustrated possible emotion specific differences across occipito-parietal (Keil et al., 2018) and separately measured occipital and parietal sites (Kujawa et al., 2012).

Due to the relationship between behavioural FEP measures and language, it may be that the aforementioned ERPs have a similar relationship with language. Indeed, previous studies across adult populations have reported emotional language effects on the P100 (Keuper et al., 2014) and N170 (Frühholz et al., 2011, Zhang et al., 2017). That is, emotional words tended to elicit heightened amplitudes when compared to neutral words. However, given the association between language and the development of emotion categories, it is possible that the relationship between general language ability and FEP may be dependent upon the emotional sensitivity of the ERP. That is, language may play a stronger role for the neurophysiological measures of FEP that show emotion effects (such as the LPP) when compared to less emotionally sensitive ERPs (such as the P100 and N170). Theoretically, this may be due to the role that language plays in FEP. Therefore, the use of face-sensitive ERPs will allow us to determine which aspects of the neural processing of facial emotions are most strongly related to language. There has been limited research investigating the relationship between language and FEP ERPs in child populations. Extant studies have generally employed small sample sizes and focused on comparing typically developing children to those with a stutter or hearing problem, without examining changes related to development (D’Hondt et al., 2017, Gu et al., 2019, Usler and Weber, 2020). Thus, it is not yet known how language may influence neurophysiological measures of FEP across development.

To our knowledge, no large-scale study has examined the development of FEP ERPs within the context of language and emotion. This research builds upon existing literature (see Bigelow et al., 2021; Cassetta et al., 2018) surrounding the relationship between language and emotion by investigating the role of language in the neural processing of facial emotion. As a result, this will provide greater insight into the time-course of these processes, and how this may change throughout development. Subsequently, this research has educational implications for the multidisciplinary approach to the promotion of linguistic and social cognitive development in neurotypical and atypically developing children. The current study aimed to investigate whether language is associated with neurophysiological indicators of FEP. It was hypothesised that the LPP amplitude would display language effects (i.e., stronger amplitude for better language skills) and emotion effects (i.e., stronger for expressive faces when compared to neutral faces), with the P100 and N170 included for comparative purposes. It was hypothesised that with increasing age, P100 and N170 latencies would shorten, and P100 and LPP amplitudes (measured separately at occipital and parietal sites) would decrease, whilst N170 amplitude would increase (i.e., become more negative).

## 2 Method

### 2.1 Participants

One hundred and fifty-six (76 female) children were initially recruited from a larger study exploring the development of social cognitive skills (Bigelow et al., 2021). Data from 42 participants were removed due to a current diagnosis (*n* = 1), English comprehension difficulties (*n* = 2), EEG recording issues (*n* = 11), or insufficient EEG data after pre-processing (*n* = 28). As shown in Table 1, this resulted in a final sample of 114 (53 female) typically developing, English-speaking children aged between 4 and 12 years, with an average age of 9.78 years (*SD* = 1.68). Male participants (*M* = 9.96, *SD* = 1.68) were slightly but not significantly older than female participants (*M* = 9.58, *SD* = 1.67), *t*(112) = 1.21, *p* = .229.

**Table 1:** Means, standard deviations and sex ratio across age groups by year.

	Age group								
	4 yrs	5 yrs	6 yrs	7 yrs	8 yrs	9 yrs	10 yrs	11 yrs	12 yrs
	*n = 1*	*n* = 3	*n* = 5	*n* = 9	*n* = 14	*n* = 28	*n* = 25	*n* = 20	*n* = 11
Mean	4.83	5.59	6.59	7.45	8.46	9.55	10.43	11.40	12.33
SD	0	0.34	0.27	0.28	0.29	0.25	0.31	0.32	0.22
F/M	1/0	1/2	3/2	3/6	9/5	14/14	13/12	5/15	5/6

*Note.* Mean = child’s mean age in years; SD = standard deviation in years; F/M = female/male sex ratio.

Of the final sample, demographic information was obtained from 107 (102 female) primary caregivers of each participant. Fifty-four percent of primary caregivers reported an annual income equal or greater than the national median income (Australian Bureau of Statistics). Sixty-eight percent of primary caregivers reported their country of birth as Australia or New Zealand, 21% as Asia, 6% as Europe/United Kingdom, 2% as North America, with the remainder not disclosing this information. Fourteen parents/guardians reported that a language other than English was spoken at home, with the most commonly reported being Korean and Cantonese. Informed consent was obtained from a parent or legal guardian, as was assent from child participants. Ethical approval for the study was obtained from Deakin University Human Research Ethics Committee (project number: 2017–065).

### 2.2 Materials

#### 2.2.1 Language

The 32-item Recalling Sentences subtest from the Clinical Evaluation of Language Fundamentals – Fourth Edition: Australian Standardisation (CELF-4; Semel et al., 2006) was used to assess broad language ability. This task measured aspects of language processing including speech production, grammatical processing, and linguistic knowledge (Conti-Ramsden et al., 2001). Recalling Sentences has high split-half reliability (*r* = 0.92), and correlates highly (*r* = 0.86) with general language measures (Klem et al., 2015, Semel et al., 2006). During the task, the child was instructed to repeat verbatim the sentence spoken to them by the examiner, with each item growing in length and complexity. The maximum possible score was 96, with higher scores indicating greater language proficiency. Raw uncorrected scores were used for all analyses.

#### 2.2.2 Facial stimuli

Facial stimuli were selected from the *Child Affective Facial Expression Stimulus Set (CAFE;* LoBue and Thrasher, 2014). This set was selected as it is ethnically and racially diverse and includes child-appropriate models (aged 2 years to 8 years; LoBue, 2014). From this set, two models were selected for the practice phase (1 female, 1 male; model numbers 6281, 6386) and four models were selected for the main task phase (2 females, 2 males; model numbers 6284, 6346, 6365, 6368). Each model depicted happy, angry, fearful, and neutral expressions. The inclusion of a neutral expression served as an ambiguous emotion that could be categorised as either positive or negative, thereby functioning as a baseline for emotion evaluation (Posamentier and Abdi, 2003). Each expression was identified accurately in 68% (for fearful expressions) to 93% (for happy expressions) of adults (LoBue and Thrasher, 2014). Similar ratings using children aged three to four years have since been replicated by LoBue et al. (2018). All stimuli were presented in colour, with models seated in front of a white background and wearing a white t-shirt to minimise any effect of clothing.

### 2.3 Facial emotion processing task

The FEP task incorporated forced-choice responses used to assess accuracy in expression identification. Participants viewed an image of a child’s face. When a blue box appeared around the picture, this signalled to the participant to press the corresponding button indicating whether the character was feeling “good” or “not good”. The option of “not good” was selected over a direct contrast such as “bad” to limit the influence that the instructions had upon the participant’s perception of an ambiguous expression such as neutrality. Refer to Supplementary Material for additional FEP task information.

**Fig. 1:**
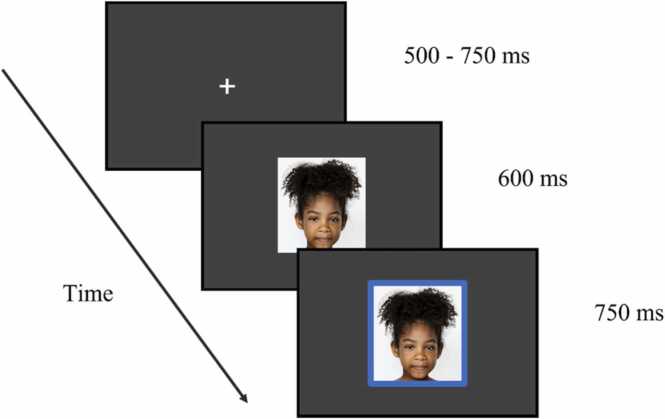
Presentation of timing during an example of a single trial. Each trial consisted of a fixation screen, the emotional stimulus, and the blue box indicating a response is required from participant. Due to copyright, stimulus pictured in the single trial is not from the CAFE stimulus set.

Source: Cookie_Studio-Freepik (2021).

Participants were seated approximately 60 cm from the 55 cm computer screen. Each trial began with a white fixation cross in the centre of a black screen randomly displayed between 500 and 750 ms. Participants were then presented with a model expressing one of four emotions: neutrality, anger, happiness, or fear, for 600 ms. Following this, a blue box appeared around the image for 750 ms. The timing of the blue box ensured that the participant’s response did not interfere with the ERP phase. If no response was recorded during the allocated 750 ms, the next trial automatically began. An example of a single trial is presented below in Fig. 1. During the practice phase, participants were able to practice the task using different stimuli to the main task, until they felt comfortable.

Participants were presented with both static and dynamic trials, although only static trials were explored in this paper to ensure the ERP reflected a single emotion. The current study used an experimental paradigm comprising 192 static trials split across four blocks, each lasting roughly three minutes. Each block contained all of the 4 characters displaying each of the 4 expressions, with the order randomised. Participants were given the option of a short break between blocks. The task was presented using E-prime 3.0 software (Psychology Software Tools, Pittsburgh, PA). While not examined in this study, before the task began, participants were provided with moral information about the character of the faces. To limit the number of moral characters the children were required to remember, it was decided that the same four faces were to be used across all FEP trials, aside from the practice task.

### 2.4 Procedure

Participants were tested in a quiet room at the child’s school or at Deakin University. The Language and EEG tasks were administered to participants on different sessions (with an average of roughly two weeks between sessions). Prior to the session beginning, written informed consent was obtained on behalf of the child from a parent/guardian. The child was also informed of all procedures in the study and agreed to participate. The parent/guardian were given a series of questionnaires and demographic information to complete. Parents/guardians were reimbursed with a $20 AUD department store voucher.

### 2.5 EEG acquisition and processing

**Fig. 2:**
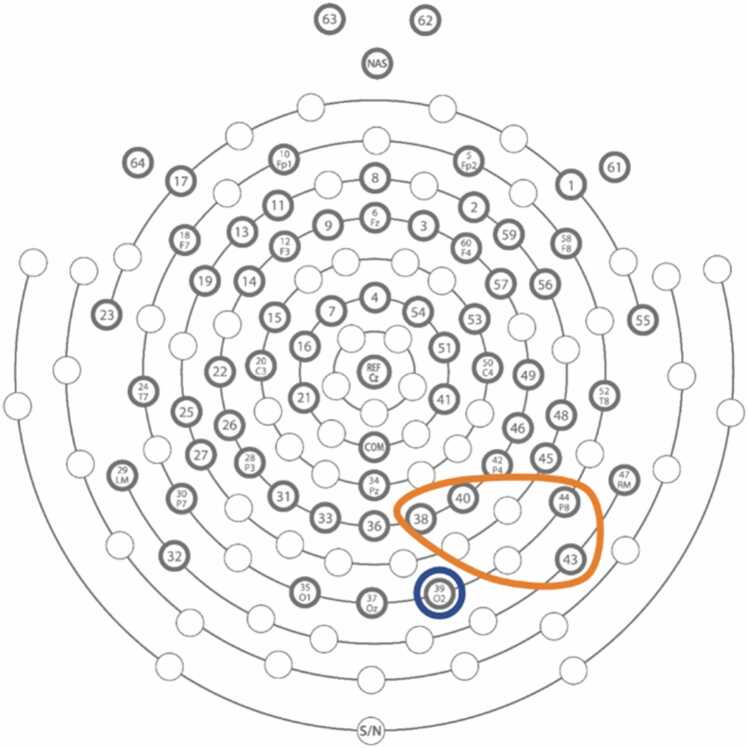
EEG electrode placement diagram. Electrode circled in blue represent P100 and LPP Occipital channels, electrodes in orange represent N170 and LPP Parietal channels.

During the FEP task, EEG was recorded using a 64-channel HydroCel Geodesic Sensor Net (Electrical Geodesics Inc, USA). Electrode placements are shown in Fig. 2. Data were acquired through NetStation 5.0 software. A sampling rate of 1000 Hz was used, with Cz as the online reference, and impedances reduced to below 50 kΩ before recording began.

Offline EEG data were processed using MATLAB 2018a (The MathWorks Inc., USA), EEGLAB 2019.0 (Delorme and Makeig, 2004), and ERPLAB 7.0.0 (Lopez-Calderon and Luck, 2014). EEG data were down-sampled to 500 Hz before a bandpass filter of 0.5–30 Hz was applied. Stimulus-locked epochs were then created from the data, from − 150 to 1000 ms referenced to stimulus onset. Bad channels were then identified using kurtosis values of ± 5 standard deviations from the mean and visual inspection. Identified channels were then replaced from surrounding channels using spherical interpolation. Epochs including data greater than ± 500 uV were removed, and data were re-referenced to the common average. Data were submitted to independent components analysis, and the ICLabel plugin (Pion-Tonachini et al., 2019) was used to identify components comprising ocular and non-ocular artefacts. These artefacts were removed to correct the EEG signal. Finally, epochs including data with a recorded amplitude greater than ± 100 uV were removed. The remaining epochs were then averaged, with 150 ms pre-stimulus onset as baseline.

EEG data were considered insufficient if less than one third of trials (< 64) for a participant remained after data cleaning. Twenty-eight participants were excluded due to insufficient EEG data, with an average age of 8.16 years (*SD* = 2.10). This suggests that the excluded participants tended to be younger than the remaining participants. Whilst important to acknowledge, this is relatively unsurprising given previous research showing that younger children persist through fewer blocks of trials (Brooker et al., 2019). Across participants, the average number of trials remaining for analysis was 140 (*SD* = 36).

**Fig. 3:**
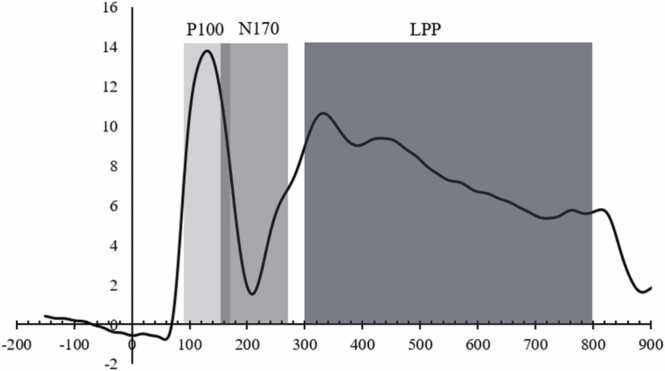
Grand averaged waveform with shaded areas representing time windows in which the P100 and N170 peaks were defined, and the time window in which the average voltage of the LPP.

Previous research (Batty and Taylor, 2006, De Haan et al., 1998, Grunewald et al., 2015, Gu et al., 2019, Luyster et al., 2019, Rossion et al., 2003, Young et al., 2017) has reported hemispheric differences in the processing of facial emotions in typically developing children. That is, there appears to be a right hemispheric specialisation reflected in heightened amplitudes when compared to the left hemisphere, as recorded across FEP ERPs including the N170 (Young et al., 2017) and LPP (Cacioppo et al., 1996, Chronaki et al., 2018, Deng et al., 2019, Usler et al., 2020; see review by Hartikainen, 2021). Indeed, this is supported by neuroimaging evidence showing that emotional faces have a right-sided effect across regions including the FFA (Aylward et al., 2005, Kanwisher et al., 1997) and pSTS (Zhu et al., 2016). Therefore, in line with previous studies (Dalrymple et al., 2011, Lee et al., 2007, Maher et al., 2016) only the data from the right hemisphere were included in analyses. Please refer to Tables S1 and S2 in Supplementary Material for left hemisphere analyses. This was supported by preliminary analyses, which indicated significant hemisphere differences between several ERPs. For the P100 component, the right occipital electrode was selected (O2), with the P100 peak defined as the most positive value within the time window of 90–170 ms. For the N170 component, right parietal electrodes were selected (P2, PO4, P8, P10), with the N170 peak defined as the most negative value within the time window of 150–270 ms. The LPP component was recorded separately at right occipital (O2) and parietal electrodes (P2, PO4, P8, P10), with the LPP peak defined as the average voltage within the time window of 300–800 ms. For comparison with studies the combined occipital and parietal electrodes, please refer to Tables S3 and S4 in Supplementary Material for analyses averaged across parietal and occipital sites. Time windows for each component were defined based on previous literature and inspection of the grand averaged waveform (as illustrated below in Fig. 3). The grand average was calculated using the average of the right channels previously mentioned, and their equivalent left hemisphere channels.

### 2.6 Data analysis

#### 2.6.1 Data transformation and screening

Prior to running the analyses, data were screened for missing values and assumption violations. Refer to Supplementary Materials for assumption testing.

#### 2.6.2 Linear mixed model

Linear mixed models were used to test hypotheses. Previous literature (Batty and Taylor, 2006, Meaux et al., 2014) has highlighted possible biological sex differences in the development of FEP ERPs. Therefore, participant biological sex was accounted for by its inclusion as a fixed effect. Emotion was included for all analyses, with neutrality serving as the reference level. This enabled the comparison of responses to expressive and neutral faces. Multiple comparisons were corrected using the false discovery rate (FDR; Benjamini and Hochberg, 1995). Each model included Participant ID as a random intercept, with Age and Language as continuous fixed effects, and Biological Sex and Emotion (anger, happiness, fear and neutrality) as categorical fixed effects to predict each FEP ERP. Interactions of Age by Emotion, Age by Language, and Emotion by Language were also entered in the models. Analyses were conducted using Stata 15 (StataCorp, 2021). See Supplementary Materials for data analysis script. Given the skewed age distribution, results were additionally run excluding the youngest participants. All findings remained. See Tables S5 and S6 in Supplementary Materials for analyses excluding youngest participants.

## 3 Results

### 3.1 Preliminary analyses

Summary results including Pearson correlations, and mean and standard deviation values for each variable are reported in Table 2. Age significantly correlated with each of the FEP ERPs (positively with N170 amplitude, negatively with all other variables). As expected (due to the use of raw language scores), Age correlated positively with Language.

**Table 2:** Pearson correlations among continuous study variables.

Variable	1	2	3	4	5	6	7
1. Age	–						
2. Language	0.50**	–					
3. P100 Amplitude	-0.21**	-0.09	–				
4. P100 Latency	-0.14*	0.02	0.10*	–			
5. N170 Amplitude	0.28**	0.16**	0.11*	0.02	–		
6. N170 Latency	-0.29**	-0.05	0.15*	0.21**	0.13*	–	
7. LPP Occipital Amplitude	-0.39**	-0.15*	0.73**	0.16**	-0.03	0.03	–
8. LPP Parietal Amplitude	-0.31**	-0.18**	0.43**	0.12*	0.31**	0.06	0.66**

*Note.* Age = child’s age in years; Raw scores used for Language.

* *p* < .05; ** *p* ≤ .001.

### 3.2 Linear mixed model analyses

Summary results for main effects and interaction effects for each of the linear mixed models are reported in Tables 3 and 4, respectively. Analyses presented are FDR corrected where necessary, unless otherwise noted.

**Table 3:** Summary of main effects.

DV	Predictor	*β*	SE	z	P > |z|	95% CI		df	*χ*^2^	P > *χ*^2^
P100 Amplitude								6	21.57	**.001**
	Age	-1.75	0.68	-2.59	**.010**	-3.07	-0.43			
	Language	0.03	0.08	0.44	.663	-0.12	0.19			
	Emotion^a^							3	5.41	.144
	Anger	0.66	0.33	2.00	.078	0.012	1.32			
	Happiness	0.65	0.33	1.94	.078	-0.01	1.30			
	Fear	0.57	0.33	1.71	.088	-0.08	1.22			
	B.S^b^							1	10.40	**.001**
	Female	-6.33	1.96	-3.23	**.001**	-10.18	2.48			
P100 Latency								6	14.53	**.024**
	Age	-2.33	0.91	-2.56	**.011**	-0.04	0.38			
	Language	0.17	0.11	1.57	.117	-4.12	-0.55			
	Emotion^a^							3	3.42	.331
	Anger	-1.49	1.07	-1.39	.248	-3.59	0.61			
	Happiness	-1.82	1.07	-1.70	.248	-3.93	0.28			
	Fear	-0.77	1.07	-0.72	.472	-2.88	1.33			
	B.S^b^							1	5.97	**.015**
	Female	-6.47	2.65	-2.44	**.015**	-11.7	-1.28			
N170 Amplitude								6	14.92	**.021**
	Age	0.83	0.29	2.86	**.004**	0.26	1.39			
	Language	0.01	0.03	0.21	.832	-0.06	0.07			
	Emotion^a^							3	2.86	.413
	Anger	-0.35	0.28	-1.24	.642	-0.89	0.20			
	Happiness	-0.18	0.28	-0.66	.762	-0.73	0.36			
	Fear	0.08	0.28	0.30	.762	-0.46	0.63			
	B.S^b^							1	0.67	.413
	Female	0.69	0.84	0.82	.413	-0.96	2.33			
N170 Latency								6	27.08	**< 0.001**
	Age	-4.74	1.06	-4.48	**< 0.001**	-6.81	-2.67			
	Language	0.20	0.12	1.66	.098	-0.04	0.45			
	Emotion^a^							3	4.20	.241
	Anger	-1.21	1.77	-0.68	.494	-4.68	2.26			
	Happiness	-2.18	1.77	-1.23	.329	-5.64	1.29			
	Fear	-3.49	1.77	-1.97	.144	-6.96	-0.03			
	B.S^b^							1	4.45	**.035**
	Female	-6.49	3.07	-2.11	**.035**	-12.51	-0.46			
LPP Occipital Amplitude								6	49.56	**< 0.001**
	Age	-1.55	0.31	-5.05	**< 0.001**	-2.16	-0.95			
	Language	0.03	0.04	0.91	.365	-0.04	0.10			
	Emotion^a^							3	15.79	**.001**
	Anger	0.57	0.27	2.14	**.033**	0.05	1.10			
	Happiness	0.62	0.27	2.32	**.032**	0.10	1.14			
	Fear	1.06	0.27	3.95	**< 0.001**	0.53	1.58			
	B.S^b^							1	7.92	**.005**
	Female	-2.51	0.89	-2.81	**.005**	-4.27	-0.76			
LPP Parietal Amplitude								6	41.89	**< 0.001**
	Age	-0.60	0.17	-3.52	**< 0.001**	-0.93	-0.27			
	Language	-0.003	0.02	-0.16	.870	-0.04	0.04			
	Emotion^a^							3	21.99	**< 0.001**
	Anger	0.53	0.20	2.67	**.008**	0.14	0.92			
	Happiness	0.62	0.20	3.13	**.003**	0.23	1.01			
	Fear	0.91	0.20	4.59	**< 0.001**	0.52	1.30			
	B.S^b^							1	4.10	**.043**
	Female	-1.00	0.50	-2.03	**.043**	-1.97	-0.03			

*Note.* Significant main effects highlighted in bold. Age = child’s age in years; B.S = Biological sex. Raw scores used for Language. FDR correction applied where appropriate.

a Emotion compared to Neutrality.

b Biological sex compared to male.

**Table 4:** Summary of interaction effects.

DV	Predictor	*β*	SE	z	P > |z|	95% CI		df	*χ*^2^	P > *χ*^2^
P100 Amplitude										
	Age × Language	-0.03	0.04	-0.80	.423	-0.10	0.04			
	Age × Emotion^a^							3	3.53	.317
	Anger	-0.36	0.23	-1.58	.348	-0.80	0.09			
	Happiness	0.02	0.23	0.09	.931	-0.43	0.46			
	Fear	-0.08	0.23	-0.35	.931	-0.53	0.36			
	Emotion^a^ × Language							3	2.49	.477
	Anger	0.01	0.03	0.44	.895	-0.04	0.06			
	Happiness	-0.004	0.03	-0.13	.895	-0.06	0.05			
	Fear	0.03	0.03	1.29	.585	-0.02	0.09			
P100 Latency										
	Age × Language	0.04	0.05	0.76	.449	-0.06	0.14			
	Age × Emotion^a^							3	10.33	**.016**
	Anger	-1.90	0.72	-2.62	**.027**	-3.31	-0.48			
	Happiness	0.12	0.72	0.16	.872	-1.30	1.53			
	Fear	-1.03	0.72	-1.43	.230	-2.45	0.38			
	Emotion^a^ × Language							3	10.56	**.014**
	Anger	0.26	0.09	3.08	**.006**	0.10	0.43			
	Happiness	0.14	0.09	1.65	.099	-0.03	0.31			
	Fear	0.21	0.09	2.42	**.024**	0.04	0.37			
N170 Amplitude										
	Age × Language	-0.004	0.02	-0.24	.809	-0.04	0.03			
	Age × Emotion^a^							3	7.60	.055
	Anger	-0.21	0.19	-1.14	.367	-0.59	0.16			
	Happiness	0.17	0.19	0.90	.367	-0.20	0.54			
	Fear	-0.30	0.19	-1.59	.336	-0.67	0.07			
	Emotion^a^ × Language							3	2.86	.414
	Anger	-0.004	0.02	-0.18	.857	-0.05	0.04			
	Happiness	-0.02	0.02	-0.94	.693	-0.06	0.02			
	Fear	0.02	0.02	0.74	.693	-0.03	0.06			
N170 Latency										
	Age × Language	0.100	0.06	1.72	.085	-0.01	0.21			
	Age × Emotion^a^							3	3.72	.294
	Anger	-2.25	1.21	-1.86	.189	-4.62	0.12			
	Happiness	-1.46	1.21	-1.21	.226	-3.84	0.91			
	Fear	-1.63	1.21	-1.34	.226	-4.00	0.75			
	Emotion^a^ × Language							3	0.62	.891
	Anger	0.03	0.14	0.20	.909	-0.25	0.31			
	Happiness	-0.02	0.14	-0.11	.909	-0.30	0.26			
	Fear	-0.08	0.14	-0.56	.909	-0.36	0.20			
LPP Occipital Amplitude										
	Age × Language	-0.01	0.02	-0.56	.574	-0.04	0.02			
	Age × Emotion^a^							3	1.51	.680
	Anger	-0.17	0.18	-0.93	.815	-0.52	0.19			
	Happiness	0.04	0.18	0.23	.815	-0.31	0.40			
	Fear	-0.05	0.18	-0.27	.815	-0.40	0.31			
	Emotion^a^ × Language							3	7.90	**.048**
	Anger	-0.03	0.02	-1.21	.342	-0.07	0.02			
	Happiness	-0.05	0.02	-2.27	.069	-0.09	-0.01			
	Fear	0.004	0.02	0.19	.845	-0.04	0.05			
LPP Parietal Amplitude										
	Age × Language	0.01	0.01	1.58	.114	-0.004	0.03			
	Age × Emotion^a^							3	0.58	.900
	Anger	-0.05	0.13	-0.38	.915	-0.31	0.21			
	Happiness	0.01	0.13	0.11	.915	-0.25	0.28			
	Fear	0.05	0.13	0.37	.915	-0.21	0.31			
	Emotion^a^ × Language							3	7.29	.063
	Anger	-0.02	0.02	-0.99	.484	-0.05	0.02			
	Happiness	-0.04	0.02	-2.40	.051	-0.07	-0.01			
	Fear	-0.002	0.02	-0.13	.895	-0.03	0.03			

*Note*. Significant interaction effects highlighted in bold. Age = child’s age in years; Raw scores used for Language. FDR correction applied where appropriate.

a Emotion compared to Neutrality.

#### 3.2.1 P100 amplitude

As hypothesised, Age had a significant impact on P100 amplitude (*z* = − 2.59, *p* = .01), indicating that as Age increased, P100 amplitude decreased. Biological sex had a significant impact on P100 amplitude (*z* = − 3.22, *p* = .001), with females showing smaller P100 amplitudes than males. Neither Emotion nor Language had a significant impact on P100 amplitude (*p*’s > 0.144). No significant interaction effects were reported for Age by Emotion, Age by Language, or Emotion by Language (*p*’s > 0.317).

#### 3.2.2 P100 Latency

As predicted, Age had a significant impact on P100 latency (*z* = − 2.56, *p* = .011), indicating that as Age increased, P100 latency decreased. Biological sex had a significant impact on P100 latency (*z* = − 2.44, *p* = .015), with females having earlier P100 latencies than males. Neither Emotion nor Language had a significant impact on P100 latency (*p*’s > 0.117).

**Fig. 4:**
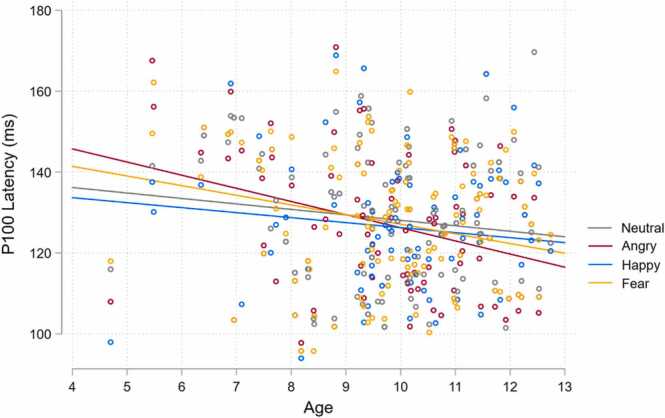
Emotion by age interaction predicting P100 latency.

The Emotion by Age interaction significantly affected P100 latency (*χ*^2^(3) = 10.33, *p* = .016). As shown in Fig. 4, P100 latency appears to decrease with Age for neutral and expressive faces. It appears that this association with Age is the strongest for anger, with a sharper decrease in latency from younger to older children. Indeed, anger was the only emotion that showed a significantly different decrease in P100 latency with Age to that of neutral faces (*z* = − 2.62, *p* = .027).

**Fig. 5:**
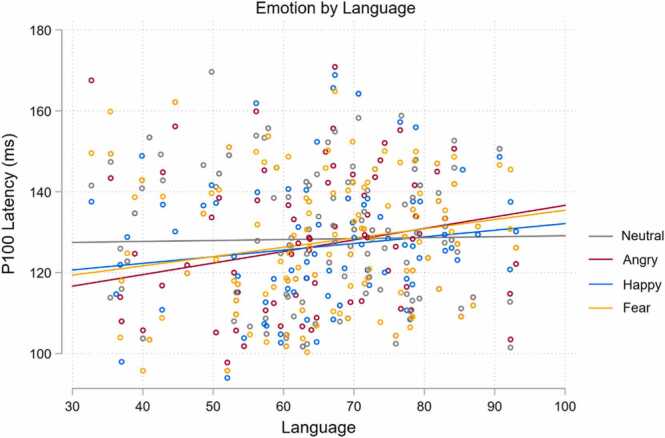
Emotion by language interaction predicting P100 latency.

Interestingly, the Emotion by Language interaction significantly affected P100 latency (*χ*^2^(3) = 10.56, *p* = .014). As shown in Fig. 5, P100 latency for each emotion, but not for neutrality, appears to increase with increasing language ability. When compared to neutrality, the P100 increase with language ability was significantly greater for anger (*z* = 3.08, *p* = .006) and fear (*z* = 2.42, *p* = .024), but not for happy (*z* = 1.65, *p* = .099). These analyses indicate that when compared to neutral faces, the P100 latency of angry and fearful facial expressions, significantly increased with increased language ability. No significant interaction effect was reported for Age by Language on P100 latency (*z* = 0.76, *p* = .450).

#### 3.2.3 N170 amplitude

In contrast with hypotheses, Age had a significant impact on N170 amplitude (*z* = 2.86, *p* = .004), indicating that as Age increased, N170 amplitude became smaller (i.e., more positive). Neither Biological sex, Emotion nor Language had a significant impact on N170 amplitude (*p*’s > 0.413). No significant interaction effects were reported for Age by Language, Age by Emotion, and Emotion by Language (*p*’s > 0.055).

#### 3.2.4 N170 latency

As predicted, Age had a significant impact on N170 latency (*z* = − 4.48, *p* < .001), indicating that as Age increased, N170 latency decreased. Biological sex also had a significant impact (*z* = − 2.11, *p* = .035), with females demonstrating shorter N170 latencies than males. In line with hypotheses, neither Emotion nor Language had a significant impact on N170 latency (*p*’s > 0.098). No significant interaction effects were reported for Age by Language, Emotion by Language, or Age by Emotion (*p*’s > 0.085).

#### 3.2.5 LPP occipital amplitude

In line with hypotheses, Age had a significant impact on LPP amplitude recorded at occipital sites (*z* = − 5.05, *p* < .001), indicating that as Age increased, LPP occipital amplitude decreased. As hypothesised, Emotion had a significant impact on LPP occipital amplitude (*χ*^2^(3) = 15.79, *p* = .001). Amplitude was stronger for happiness (*z* = 2.32, *p* = .032), fear (*z* = 3.95, *p* < .001), and anger (*z* = 2.13, *p* = .033) expressive faces when compared to neutral faces. Biological sex had a significant impact on LPP occipital amplitude (*z* = − 2.81, *p* = .005), with females showing smaller LPP occipital amplitudes than males. In contrast with hypotheses, Language did not have a significant impact on LPP occipital amplitude (*z* = 0.91, *p* = .365).

**Fig. 6:**
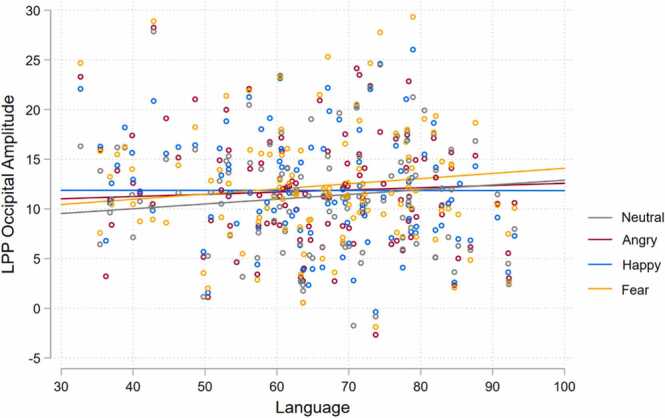
Emotion by language interaction predicting LPP occipital amplitude.

The Emotion by Language interaction significantly affected LPP occipital amplitude (*χ*^2^(3) = 7.90, *p* = .048). As shown in Fig. 6, it appears that amplitude to angry, neutral, and fearful faces increases with language ability, while the amplitude to happiness does not change with language ability. Whilst not surviving FDR correction, analyses indicated that happiness (*z* = − 2.27, *p* = .069) appeared to be trending towards a different association between LPP amplitude and language skills when compared to neutral faces. No significant interaction effects were reported for Age by Language, and Emotion by Age, (*p*’s > 0.574).

#### 3.2.6 LPP parietal amplitude

As predicted, Age had a significant impact on LPP amplitude recorded at parietal sites (*z* = − 3.52, *p* < .001), indicating that as Age increased, LPP parietal amplitude decreased. In line with hypotheses, Emotion had a significant impact on LPP parietal amplitude (*χ*^2^(3) = 21.99, *p* < .001), indicating that amplitude was stronger for expressive faces when compared to neutral faces. Amplitude was stronger for happiness (*z* = 3.13, *p* = .003), fear (*z* = 4.59, *p* < .001), and anger (*z* = 2.67, *p* = .008) expressive faces when compared to neutral faces. Biological sex had a significant impact on LPP amplitude (*z* = − 2.03, *p* = .043). In contrast with hypotheses, Language did not have a significant impact on LPP parietal amplitude (*z* = −0.16, *p* = .870). No significant interaction effects were reported for Age by Language, Age by Emotion, and Language by Emotion (*p*’s > 0.063).

## 4 Discussion

This study examined the relationship between age, language and FEP ERPs during early to middle childhood. As predicted, as age increased, P100 amplitude and latency decreased, N170 latency decreased, and LPP amplitude decreased. In contrast with hypotheses, N170 amplitude tended to decrease (i.e., become more positive), with increasing age. In line with hypotheses, no main effects of emotion or language were found across the early ERP components (P100 and N170). Emotion effects were present at both LPP occipital and parietal sites, with expressive faces eliciting larger amplitudes than neutral faces. In line with hypotheses, language appeared to interact with emotion for LPP amplitude measured at occipital sites, however, no interaction effects were found for LPP amplitude measured at parietal sites. Surprisingly, an interaction effect of emotion by language was observed for P100 latency.

### 4.1 Facial emotion processing across development

In line with previous research (Batty and Taylor, 2006, Deng et al., 2019, Keil et al., 2018, MacNamara et al., 2016), significant age effects were observed for P100, N170 and LPP. That is, older children tended to have shorter P100 and N170 latencies and reduced P100 and LPP amplitudes than younger children. Comparatively, LPP occipital amplitudes tended to be stronger and to decrease more strongly with age, than parietal amplitudes. This supports previous research (Kujawa et al., 2012) suggesting that LPP amplitude is initially strongest at occipital sites in earlier childhood, before gradually moving towards parietal sites during later childhood. This suggests that early neurophysiological measures of FEP are continuing to undergo developmental changes between 4 and 12 years. These developmental findings may indicate a gradually developing facial processing specialisation, or may reflect general improvements in early visual processing efficiency resulting from synaptic pruning (MacNamara et al., 2016).

In contrast with previous research (Battaglia et al., 2007; Chronaki et al., 2018; Hoyniak et al., 2019), N170 amplitude was found to decrease (i.e., become more positive) with increasing age. This result is, however, in line with previous research (Batty and Taylor, 2006, Meaux et al., 2014) illustrating smaller N170 amplitudes with increasing age during early to middle childhood. It is possible that the relationship between age and N170 amplitude is non-linear, as has been suggested in previous research (Taylor et al., 2004). N170 amplitude may initially increase in strength (i.e., becoming more negative) in early childhood, before decreasing in strength during middle childhood, and finally increasing in strength in adolescence. Therefore, it is possible that the age range included in this study (mean of 9.78 years) may capture only part of this non-linear relationship, and may fail to show the subsequent increase in N170 amplitude with increasing age as previously shown in adolescent samples (Batty and Taylor, 2006). Theoretically, changes in N170 amplitude may be illustrative of a transformation in FEP specificity. That is, the transformation from the featural processing of facial emotions to the configural processing of facial emotions (Aylward et al., 2005).

### 4.2 The emotional sensitivity of facial processing across development

In support of previous literature (Keil et al., 2018, Kujawa et al., 2012, Usler et al., 2020), LPP amplitude displayed main effects of emotion at both occipital and parietal sites. Specifically, amplitudes were stronger for expressive faces when compared to neutral faces. This indicates that children aged 4–12 years process emotional and neutral facial stimuli differently, between 300 and 800 ms post stimulus onset. It is possible that the heightened amplitudes towards expressive faces may be due to the additional allocation of cognitive resources towards expressive stimuli (Usler et al., 2020). Interestingly, the LPP was the only ERP to show emotional effects extending to positive expressions (i.e., happiness). Overall, results suggest that emotion effects are most consistent after 300 ms.

Results suggested some degree of emotional sensitivity for the P100. An Emotion by Age interaction was observed for P100 latency, with angry faces appearing to decrease most substantially with age. This suggests that children may develop an ability to preferentially attend towards angry faces. Indeed, this has been suggested in previous studies showing that infants have a shorter reaction time to angry faces when compared to happy faces (LoBue and Deloache, 2010). This finding has been extended to include preferential attention to threatening *stimuli* (such as spiders or snakes), rather than exclusively threatening *faces* (Öhman et al., 2001). In line with some previous research (Curtis and Cicchetti, 2011, O’Connor et al., 2005, Todd et al., 2008, Usler and Weber, 2020), no emotion effects were found for the N170 in children aged 4–12 years. This suggests that whilst the N170 is considered to be a *face*-sensitive ERP (Gao et al., 2019, Itier and Taylor, 2004), the N170 may not be an *emotion-*sensitive ERP in children. However, these results contrast with some research finding the N170 latency (D’Hondt et al., 2017) and amplitude (Batty and Taylor, 2006) to be emotionally sensitive. Interestingly, N170 amplitude emotion effects were only observed by Batty and Taylor (2006) for individuals aged 14–15 years. Furthermore, emotional sensitivity of the N170 amplitude has been reported in a previous meta-analysis by Hinojosa et al. (2015) in adults. Therefore, it is possible that the emotional sensitivity of the N170 may not yet be developed in children aged 4–12 years.

### 4.3 The interactive role of language and emotion during facial processing

Interaction effects of Emotion by Language were observed for P100 latency and LPP Occipital amplitude across children aged 4–12 years. Both the P100 and LPP Occipital amplitude were measured from the same occipital electrode. Interestingly, the emotions tending to drive these interactions were different. The change in P100 latency was strongest for anger and fear in comparison to neutral, with better language skills associated with longer latencies in these negative emotional expressions. This is perhaps counterintuitive, as longer latencies (suggesting slower processing) were associated with stronger language ability. For LPP Occipital amplitude, results appear to be trending towards significance, suggesting that happy expressions when compared to neutral may not elicit a heightened amplitude with increasing language skills, with both negative and neutral expressions appearing to display a positive association with LPP amplitude. However, given the lack of significance, results must be interpreted with extreme caution. This may signal that language ability was not associated with the processing of happy facial expressions to the extent of angry or fearful expressions when compared to neutrality. This may suggest that the processing of happy faces may be developmentally invariant. However, the processing of negative or ambiguous emotions may require more elaborate interpretation and processing, thus more strongly relying on language (Kisley et al., 2007, Tottenham et al., 2013).

Alternatively, it is possible that language ability may influence a negative attentional bias. That is, an attentional bias towards negative or ambiguous expressions may, in part, be driven by the existence of more negative emotion words when compared to positive emotion words in the English language (Vaish et al., 2008). This might also contribute to the apparent delay in P100 processing. Conversely, it is possible that results reflect the development of negative and positive emotion words. For example, previous studies suggest that positive words are often learnt by children at an earlier age than negative words (Baron-Cohen et al., 2010, Li and Yu, 2015). Therefore, the role of language in FEP may suggest developmental variation in the learning of negative words.

Collectively, the LPP and P100 emotion by language interactions support constructionist theories of FEP development (Barrett et al., 2007, Hoemann et al., 2019, Lindquist et al., 2015). That is, language may assist in the construction of emotion categories, as measured through FEP during early-to-middle childhood. Additionally, the role of language appears to vary across emotion. For example, language may play a greater role in the predictive processing of expressive emotion categories during FEP, such as ‘anger’, when compared to a non-expressive emotion category, such as ‘neutral’. Interestingly, language appeared to play a role in FEP despite the lack of emotion words during the task. Therefore, this study provides novel insight into the role of language in constructionist theories of emotion processing.

Overall, the interaction effects of emotion by language suggest that language plays a role across early neurophysiological indicators of FEP in children aged 4–12 years, but that it is dependent on the facial expression. Given that the behavioural association between language and FEP is relatively strong, it appears that the current findings suggest a more nuanced association with neural processes underpinning FEP.

### 4.4 Contrasts between the role of language in neurophysiological and behavioural measures of facial emotion processing

Language was not an independent predictor of neurophysiological measures of FEP related skills in children aged 4–12 years. Despite previous behavioural findings implicating language and FEP (Astington and Jenkins, 1999; Beck et al., 2012; Bigelow et al., 2021; Gallant et al., 2020), no main effect was found between language and neurophysiological measures of FEP. This indicates that any association with language is unlikely to be broadly linked to the early stages of FEP in children. It is possible that the relationship between language and electrophysiological measures of FEP may strengthen with age, and therefore may not be evident in children aged 4–12 years, or indeed limited to specific emotional expressions (e.g., negative and/or ambiguous). Alternatively, it is possible that the complexity of the relationship between language and FEP ERPs prevented it from being observed in this study.

Considering the lack of a broader association between language and FEP ERPs, it may be that electrophysiological indicators of FEP may capture aspects of emotional processing not related to language, or lack the sensitivity necessary to detect a relationship between FEP and language. That is, whilst behavioural measures of FEP have previously been shown to correlate strongly with language (see Bigelow et al., 2021; Cassetta et al., 2018), the technique of EEG may lack the ability to tap into fine-grained brain processes required to determine what is underlying this association. Alternatively, it may be that language largely plays a role in assisting FEP beyond 1000 ms. Indeed, this may assist in explaining previous behavioural FEP findings, since behavioural responses extended beyond 1000 ms post stimulus onset. Conversely, it is possible that any effect on FEP may only be evident when language assisting in processing the emotional stimuli is present. Indeed, recent research with adult populations suggests that language may serve as a context for emotion perception (Doyle et al., 2021) and FEP (Liu et al., 2019). Therefore, language may influence neurophysiological measures of FEP when stimuli have been presented with contextually relevant language capable of providing additional insight into the emotional state of the stimulus. It is also possible that behavioural associations between FEP and language are limited to specific emotional expressions, but associations with specific emotions are often not examined.

While not a focus of this study, it is important to acknowledge how biological sex may influence neurophysiological measures of FEP. Across participants, females tended to have significantly shorter latencies and smaller amplitudes across ERP components when compared to males. These results support previous literature (Batty and Taylor, 2006) and may reflect a female advantage at processing non-verbal cues and an emotional sensitivity necessary for survival (McClure, 2000).

### 4.5 Limitations

It is important to note that more negative facial expressions were used in this study, whilst only one positive facial expression was used. Therefore, it is possible that results may have been influenced by this imbalance. It is recommended that future studies incorporate additional positive expressions such as awe and pride to address such limitations. A limited number of children under the age of six years participated in this study. Therefore, this skewed distribution may not reflect early childhood development, and requires further exploration in future studies. It is possible that results may be affected by the electrode sites used in this study. Given that neural activity was recorded at the same electrode sites for participants aged 4–12 years, it is possible that any posterior-anterior developmental patterns were overlooked. Therefore, the emotion and language effects may be more stable at occipital sites, whilst activity at parietal sites continues to develop into adolescence (Chronaki et al., 2018, Kujawa et al., 2012). This may clarify why differences were observed across LPP occipital and parietal amplitudes, with LPP activity moving towards parietal sites in adolescence. Results from this study highlight the importance of measuring LPP separately at parietal and occipital sites when investigating child populations. It is recommended that future studies explore facial emotion identification accuracy to determine any association with language across development.

### 4.6 Conclusions and future research

This study provides evidence to support the ongoing development of FEP ERPs during children aged 4–12 years. Findings from this study indicate that language seems to play an interactive role in for the processing of certain emotions across early and later ERPs, with associations seen at both P100 (latency) and LPP (amplitude) for specific expressions. Interestingly, the relationship between language and behavioural measures of FEP are not reflected to the same degree a neurophysiological level. To gain a greater understanding on the role of language in expressive FEP, it is recommended that future research consider the association between language and FEP ERPs. Overall results suggest that expressive FEP undergoes a distinct developmental pattern when compared to the processing of neutral facial stimuli during early to middle childhood. This study highlights the need for additional research to consider the role of language in FEP. Consequently, this has potential implications for the development of strategies to improve wider social cognitive functioning. Future research may explore the role of language in the development of more complex emotions, as well as considering the contextual effect of language on FEP.

## Declaration of Competing Interest

The authors declare that they have no known competing financial interests or personal relationships that could have appeared to influence the work reported in this paper.

## Data Availability

The data that support the findings of this study are available upon request.

## References

[bib1] Apicella F., Sicca F., Federico R.R., Campatelli G., Muratori F. (2013). Fusiform gyrus responses to neutral and emotional faces in children with autism spectrum disorders: a high density ERP study. Behav. Brain Res..

[bib2] Astington J.W., Jenkins J.M. (1999). A longitudinal study of the relation between language and theory-of-mind development. Dev. Psychol..

[bib3] Australian Bureau of Statistics (2017–2018 financial year). Household Income and Wealth, Australia, ABS Website, accessed June 2021.

[bib4] Aylward E.H., Park J.E., Field K.M., Parsons A.C., Richards T.L., Cramer S.C., Meltzoff A.N. (2005). Brain activation during face perception: evidence of a developmental change. J. Cogn. Neurosci..

[bib5] Bahn D., Vesker M., Schwarzer G., Kauschke C. (2021). A multimodal comparison of emotion categorization abilities in children with developmental language disorder. J. Speech Lang. Hear. Res..

[bib6] Baron-Cohen S., Golan O., Wheelwright S., Granader Y., Hill J. (2010). Emotion word comprehension from 4 to 16 years old: a developmental survey. Front. Evol. Neurosci..

[bib7] Barrett L.F., Lindquist K.A., Gendron M. (2007). Language as context for the perception of emotion. Trends Cogn. Sci..

[bib8] Battaglia M., Michelini G., Pezzica E., Ogliari A., Fagnani C., Stazi M.A., Bertoletti E., Scaini S. (2017). Shared genetic influences among childhood shyness, social competences, and cortical responses to emotions. J. Exp. Child Psychol..

[bib9] Battaglia M., Zanoni A., Giorda R., Pozzoli U., Citterio A., Beri S., Ogliari A., Nobile M., Marino C., Molteni M. (2007). Effect of the catechol-O-methyltransferase val158met genotype on children’s early phases of facial stimuli processing. Genes, Brain and Behavior.

[bib10] Batty M., Meaux E., Wittemeyer K., Rogé B., Taylor M.J. (2011). Early processing of emotional faces in children with autism: An event-related potential study. Journal of Experimental Child Psychology.

[bib11] Batty M., Taylor M.J. (2006). The development of emotional face processing during childhood. Dev. Sci..

[bib12] Beck L., Kumschick I.R., Eid M., Klann-Delius G. (2012). Relationship between language competence and emotional competence in middle childhood. Emotion.

[bib13] Benjamini Y., Hochberg Y. (1995). Controlling the false discovery rate: a practical and powerful approach to multiple testing. J. R. Stat. Soc. B.

[bib14] Bentin S., Allison T., Puce A., Perez E., McCarthy G. (1996). Electrophysiological studies of face perception in humans. J. Cogn. Neurosci.

[bib15] Bigelow F.J., Clark G.M., Lum J.A.G., Enticott P.G. (2021). The mediating effect of language on the development of cognitive and affective theory of mind. J. Exp. Child Psychol..

[bib16] Brooker R.J., Bates J.E., Buss K.A., Canen M.J., Dennis-Tiwary T.A., Gatzke-Kopp L.M., Hoyniak C., Klein D.N., Kujawa A., Lahat A. (2019). Conducting event-related potential (ERP) research with young children. J. Psychophysiol..

[bib17] Brooks J.A., Freeman J.B. (2018). Conceptual knowledge predicts the representational structure of facial emotion perception. Nat. Hum. Behav..

[bib18] Brooks J.A., Shablack H., Gendron M., Satpute A.B., Parrish M.H., Lindquist K.A. (2017). The role of language in the experience and perception of emotion: a neuroimaging meta-analysis. SCAN.

[bib19] Cacioppo J.T., Crites S.L., Gardner W.L. (1996). Attitudes to the right: evaluative processing is associated with lateralized late positive event-related brain potentials. Pers. Soc. Psychol. Bull..

[bib20] Cassetta B.D., Pexman P.M., Goghari V.M. (2018). Cognitive and affective theory of mind and relations with executive functioning in middle childhood. Merrill-Palmer Q..

[bib21] Chronaki G., Broyd S.J., Garner M., Benikos N., Thompson M.J.J., Sonuga-Barke E.J.S., Hadwin J.A. (2018). The moderating effect of self-reported state and trait anxiety on the late positive potential to emotional faces in 6-11-year-old children. Front. Psychol..

[bib22] Conte S., Richards J.E., Guy M.W., Xie W., Roberts J.E. (2020). Face-sensitive brain responses in the first year of life. NeuroImage.

[bib23] Conti-Ramsden G., Botting N., Faragher B. (2001). Psycholinguistic markers for specific language impairment (SLI). J. Child Psychol. Psychiat..

[bib24] Cookie_Studio, 2021. Portrait of cute little boy with ginger hair pointing with fingers of both hands on white t-shirt and smiling [Photograph]. Freepik. 〈https://www.freepik.com/free-photo/portrait-cute-little-boy-with-ginger-hair-pointing-with-fingers-both-hands-white-t-shirt-smiling_9118493〉.

[bib25] Curtis W.J., Cicchetti D. (2011). Affective facial expression processing in young children who have experienced maltreatment during the first year of life: An event-related potential study. Dev. Psychopathol..

[bib26] D’Hondt F., Lassonde M., Thebault-Dagher F., Bernier A., Gravel J., Vannasing P., Beauchamp M.H. (2017). Electrophysiological correlates of emotional face processing after mild traumatic brain injury in preschool children. CABN.

[bib27] Dalrymple K.A., Oruc I., Duchaine B., Pancaroglu R., Fox C.J., Iaria G., Handy T.C., Barton J.J.S. (2011). The anatomic basis of the right face-selective N170 in acquired prosopagnosia: a combined ERP/fMRI study. Neuropsychologia.

[bib28] De Haan M., Nelson C.A., Gunnar M.R., Tout K.A. (1998). Hemispheric differences in brain activity related to the recognition of emotional expressions by 5-year-old children. Dev. Neuropsychol..

[bib29] De Haan M., Pascalis O., Johnson M.H. (2002). Specialization of neural mechanisms underlying face recognition in human infants. J. Cogn. Neurosci..

[bib30] Delorme A., Makeig S. (2004). EEGLAB: an open-source toolbox for analysis of single- trial EEG dynamics. J. Neurosci. Methods.

[bib31] Deng X., Sang B., Ku Y., Sai L. (2019). Age-related differences in the late positive potential during emotion regulation between adolescents and adults. Sci. Rep..

[bib32] Dennis T.A., Hajcak G. (2009). The late positive potential: a neurophysiological marker for emotion regulation in children. J. Child Psychol. Psychiatry.

[bib33] Doyle C.M., Gendron M., Lindquist K.A. (2021). Language is a unique context for emotion perception. Affect. Sci..

[bib34] Eimer M., Holmes A. (2007). Event-related brain potential correlates of emotional face processing. Neuropsychologia.

[bib35] Frühholz S., Jellinghaus A., Herrmann M. (2011). Time course of implicit processing and explicit processing of emotional faces and emotional words. Biol. Psychol..

[bib36] Fugate J.M.B., O’Hare A.J., Emmanuel W.J.S. (2018). Emotion words: facing change. J. Exp. Soc. Psychol..

[bib37] Gallant C.M.M., Lavis L., Mahy C.E.V. (2020). Developing an understanding of others’ emotional states: Relations among affective theory of mind and empathy measures in early childhood. Br. J. Psychol..

[bib38] Gao C., Conte S., Richards J.E., Xie W., Hanayik T. (2019). The neural sources of N170: Understanding timing of activation in face-selective areas. Psychophysiology.

[bib39] Griffiths S., Goh S.K.Y., Norbury C.F., Gooch D., Baird G., Charman T., Pickles A., Simonoff E., Bishop D. (2020). Early language competence, but not general cognitive ability, predicts children’s recognition of emotion from facial and vocal cues. PeerJ.

[bib40] Grossmann T., Johnson M.H. (2007). The development of the social brain in human infancy. Eur. J. Neurosci..

[bib41] Grunewald M., Stadelmann S., Brandeis D., Jaeger S., Matuschek T., Weis S., Kalex V., Hiemisch A., von Klitzing K., Döhnert M. (2015). Early processing of emotional faces in a go/nogo task: lack of N170 right-hemispheric specialisation in children with major depression. J. Neural. Transm..

[bib42] Gu H., Chen Q., Xing X., Zhao J., Li X. (2019). Facial emotion recognition in deaf children: evidence from event-related potentials and event-related spectral perturbation analysis. Neurosci. Lett..

[bib43] Hartikainen K.M. (2021). Emotion-attention interaction in the right hemisphere. Brain Sci..

[bib44] Herba C.M., Landau S., Russell T., Ecker C., Phillips M.L. (2006). The development of emotion-processing in children: effects of age, emotion, and intensity. J. Child Psychol. Psychiatry.

[bib45] Herrmann M.J., Ehlis A.C., Ellgring H., Fallgatter A.J. (2005). Early stages (P100) of face perception in humans as measured with event-related potentials (ERPs). J. Neural Transm..

[bib46] Hinojosa J.A., Mercado F., Carretié L. (2015). N170 sensitivity to facial expression: a meta-analysis. Neurosci. Biobehav. Rev..

[bib47] Hoemann K., Xu F., Barrett L.F. (2019). Emotion words, emotion concepts, and emotional development in children: a constructionist hypothesis. Dev. Psychol..

[bib48] Hoyniak C.P., Bates J.E., Yang C.L., Darcy I, Fontaine N.M.G. (2019). Diminished Neural Responses to Emotionally Valenced Facial Stimuli: A Potential Biomarker for Unemotional Traits in Early Childhood. Child Psychiatry and Human Development.

[bib49] Hua M., Han Z.R., Chen S., Yang M., Zhou R., Hu S. (2014). Late positive potential (LPP) modulation during affective picture processing in preschoolers. Biol. Psychol..

[bib50] Itier R.J., Taylor M.J. (2004). Effects of repetition and configural changes on the development of face recognition processes. Dev. Sci..

[bib51] Jessen S., Grossmann T. (2016). The developmental emergence of unconscious fear processing from eyes during infancy. J. Exp. Child Psychol..

[bib52] Kanwisher N., McDermott J., Chun M.M. (1997). The fusiform face area: a module in human extrastriate cortex specialized for face perception. J. Neurosci..

[bib53] Kårstad S.B., Wichstrøm L., Reinfjell T., Belsky J., Berg-Nielsen T.S. (2015). What enhances the development of emotion understanding in young children? A longitudinal study of interpersonal predictors. Br. J. Psychol..

[bib54] Keil V., Uusberg A., Blechert J., Tuschen-Caffier B., Schmitz J. (2018). Facial gender but not emotion distinguishes neural responses of 10- to 13-year-old children with social anxiety disorder from healthy and clinical controls. Biol. Psychol..

[bib55] Keuper K., Zwanzger P., Nordt M., Eden A., Laeger I., Zwitserlood P., Kissler J., Junghöfer M., Dobel C. (2014). How “love” and “hate” differ from “sleep”: using combined electro/magnetoencephalographic data to reveal the sources of early cortical responses to emotional words. Hum. Brain Mapp..

[bib56] Kisley M.A., Wood S., Burrows C.L. (2007). Looking at the sunny side of life. Psychol. Sci..

[bib57] Klem M., Melby-Lervåg M., Hagtvet B., Lyster S.A.H., Gustafsson J.E., Hulme C. (2015). Sentence repetition is a measure of children’s language skills rather than working memory limitations. Dev. Sci..

[bib58] Kotsoni E., De Haan M., Johnson M.H. (2001). Categorical perception of facial expressions by 7-month-old infants. Perception.

[bib59] Kujawa A., Klein D.N., Hajcak G. (2012). Electrocortical reactivity to emotional images and faces in middle childhood to early adolescence. Dev. Cogn. Neurosci..

[bib60] Lee S.H., Kim E.Y., Kim S.R., Im W.Y., Seo H.S., Han S.W., Park Y.M., Kim H. (2007). Facial affect perception and event-related potential N170 in schizophrenia: a preliminary study. Clin. Psychopharmacol. Neurosci..

[bib61] Leppänen J.M., Moulson M.C., Vogel-Farley V.K., Nelson C.A. (2007). An ERP study of emotional face processing in the adult and infant brain. Child Dev..

[bib62] Li Y., Yu D. (2015). Development of emotion word comprehension in Chinese children from 2 to 13 years old: Relationships with valence and empathy. PLoS One.

[bib63] Lindquist K.A., Gendron M. (2013). What’s in a word? Language constructs emotion perception. Emot. Rev..

[bib64] Lindquist K.A., Satpute A.B., Gendron M. (2015). Does language do more than communicate emotion?. Curr. Dir. Psychol. Sci..

[bib65] Liu S., Tan Q., Han S., Li W., Wang X., Gan Y., Xu Q., Zhang X., Zhang L. (2019). The language context effect in facial expressions processing and its mandatory characteristic. Sci. Rep..

[bib66] LoBue V. (2014). The Child Affective Facial Expression (CAFE) set. Databrary.

[bib67] LoBue V., Deloache J.S. (2010). Superior detection of threat-relevant stimuli in infancy. Dev. Sci..

[bib68] LoBue V., Baker L., Thrasher C. (2018). Through the eyes of a child: preschoolers’ identification of emotional expressions from the child affective facial expression (CAFE) set. Cogn. Emot..

[bib69] LoBue V., Thrasher C. (2014). The Child Affective Facial Expression (CAFE) set: validity and reliability from untrained adults. Front. Psychol..

[bib70] Lopez-Calderon J., Luck S.J. (2014). ERPLAB: an open-source toolbox for the analysis of event-related potentials. Front. Hum. Neurosci..

[bib71] Luyster R.J., Bick J., Westerlund A., Nelson C.A. (2019). Testing the effects of expression, intensity and age on emotional face processing in ASD. Neuropsychologia.

[bib72] MacNamara A., Vergés A., Kujawa A., Fitzgerald K.D., Monk C.S., Phan K.L. (2016). Age-related changes in emotional face processing across childhood and into young adulthood: evidence from event-related potentials. Dev. Psychobiol..

[bib73] Maher S., Mashhoon Y., Ekstrom T., Lukas S., Chen Y. (2016). Deficient cortical face-sensitive N170 responses and basic visual processing in schizophrenia. Schizophr. Res..

[bib74] McClure E.B. (2000). A meta-analytic review of sex differences in facial expression processing and their development in infants, children, and adolescents. Psychol. Bull..

[bib75] Meaux E., Hernandez N., Carteau-Martin I., Martineau J., Barthélémy C., Bonnet-Brilhault F., Batty M. (2014). Event-related potential and eye tracking evidence of the developmental dynamics of face processing. Eur. J. Neurosci..

[bib76] Miki K., Watanabe S., Teruya M., Takeshima Y., Urakawa T., Hirai M., Honda Y., Kakigi R. (2011). The development of the perception of facial emotional change examined using ERPs. Clinical Neurophysiology.

[bib77] Nook E.C., Lindquist K.A., Zaki J. (2015). A new look at emotion perception: concepts speed and shape facial emotion recognition. Emotion.

[bib78] Nook E.C., Sasse S.F., Lambert H.K., McLaughlin K.A., Somerville L.H. (2017). Increasing verbal knowledge mediates development of multidimensional emotion representations. Nat. Hum. Behav..

[bib79] O’Connor K., Hamm J.P., Kirk I.J. (2005). The neurophysiological correlates of face processing in adults and children with Asperger’s syndrome. Brain Cogn..

[bib80] Öhman A., Flykt A., Esteves F. (2001). Emotion drives attention: detecting the snake in the grass. J. Exp. Psychol. Gen..

[bib81] Peltola M.J., Leppänen J.M., Mäki S., Hietanen J.K. (2009). Emergence of enhanced attention to fearful faces between 5 and 7 months of age. SCAN.

[bib82] Pion-Tonachini L., Kreutz-Delgado K., Makeig S. (2019). ICLabel: an automated electroencephalographic independent component classifier, dataset, and website. NeuroImage.

[bib83] Pons F., Lawson J., Harris P.L., De Rosnay M. (2003). Individual differences in children’s emotion understanding: Effects of age and language. Scand. J. Psychol..

[bib84] Posamentier M.T., Abdi H. (2003). Processing faces and facial expressions. Neuropsychol. Rev..

[bib85] Rossion B., Joyce C.A., Cottrell G.W., Tarr M.J. (2003). Early lateralization and orientation tuning for face, word, and object processing in the visual cortex. NeuroImage.

[bib86] Ruba A.L., Repacholi B.M. (2020). Do preverbal infants understand discrete facial expressions of emotion?. Emot. Rev..

[bib87] Schupp H.T., Junghöfer M., Öhman A., Weike A.I., Stockburger J., Hamm A.O. (2004). The facilitated processing of threatening faces: an ERP analysis. Emotion.

[bib88] Semel E., Wiig E., Secord W. (2006).

[bib89] Stata 15 (StataCorp) (2021).

[bib90] Strand P.S., Downs A., Barbosa-Leiker C. (2016). Does facial expression recognition provide a toehold for the development of emotion understanding?. Dev. Psychol..

[bib91] Taylor M.J., Batty M., Itier R.J. (2004). The faces of development: A review of early face processing over childhood. Journal of Cognitive Neuroscience.

[bib92] Todd R.M., Lewis M.D., Meusel L.A., Zelazo P.D. (2008). The time course of social-emotional processing in early childhood: ERP responses to facial affect and personal familiarity in a go-nogo task. Neuropsychologia.

[bib93] Tottenham N., Phuong J., Flannery J., Gabard-Durnam L., Goff B. (2013). A negativity bias for ambiguous facial-expression valence during childhood: converging evidence from behavior and facial corrugator muscle responses. Emotion.

[bib94] Usler E., Foti D., Weber C. (2020). Emotional reactivity and regulation in 5- to 8-year-old children: an ERP study of own-age face processing. Int. J. Psychophysiol..

[bib95] Usler E.R., Weber C. (2020). Emotion processing in children who do and do not stutter: an ERP study of electrocortical reactivity and regulation to peer facial expressions. J. Fluency Disord..

[bib96] Vaish A., Grossmann T., Woodward A. (2008). Not all emotions are created equal: the negativity bias in social-emotional development. Psychol. Bull..

[bib97] Widen S.C., Russell J.A. (2003). A closer look at preschoolers’ freely produced labels for facial expressions. Dev. Psychol..

[bib98] Xie W., McCormick S.A., Westerlund A., Bowman L.C., Nelson C.A. (2019). Neural correlates of facial emotion processing in infancy. Dev. Sci..

[bib99] Young A., Luyster R.J., Fox N.A., Zeanah C.H., Nelson C.A. (2017). The effects of early institutionalization on emotional face processing: evidence for sparing via an experience-dependent mechanism. Br. J. Psychol..

[bib100] Zhang J., Wu C., Meng Y., Yuan Z. (2017). Different neural correlates of emotion-label words and emotion-laden words: an ERP study. Front. Hum. Neurosci..

[bib101] Zhu X., Bhatt R.S., Joseph J.E. (2016). Pruning or tuning? Maturational profiles of face specialization during typical development. Brain Behav..

