# Supplementary material for: Facial emotion processing and language during early-to-middle childhood development: An event related potential study
Source: Dev Cogn Neurosci. 2021 Dec 17;53:101052. doi: 10.1016/j.dcn.2021.101052 (PMC8717415; doi:10.1016/j.dcn.2021.101052)
Supplement: Supplementary file 1 — Supplementary material [file mmc1.docx]

**Supplementary material**

**Facial emotion processing task**

*Familiarisation phase.* Participants were informed that they would learn information about four characters (2 female), and that they needed to remember if a character behaved well or not well. The presentations of each pair of characters included a moral vignette. Participants were presented with text and audio soundtrack (in an adult male voice) describing two short stories, each accompanied by two of the four characters expressing neutrality. As part of a study investigating the influence of moral information, each vignette contained an introductory statement, and two (one physical and one psychological) examples of harm/care behaviours. One pair were accompanied by a vignette depicting the characters engaging in positive behaviour (i.e., helping others), whilst the other pair were accompanied by a vignette depicting the characters engaging in negative behaviour (i.e., bullying others).

Whilst character pairings and names remained constant, counterbalancing within moral conditions were implemented to reduce the introduction of confounding variables. Given the scope of this paper, the moral component is not expanded upon further.

To ensure participants remembered how each of the characters had behaved in the vignette, participants were tested. Participants were presented with a screen including each of the four characters expressing neutrality. Participants were then required to indicate which of the characters behaved positively, and then negatively. This was achieved by the participant either pointing to the character or referring to the character by name. The order in which testing occurred was counterbalanced. If the participant responded incorrectly, they were shown the vignettes again and were asked to try again. This continued until the participant remembered each character’s story.

**Table S1**

*Summary of Main Effects for Left Hemisphere and Averaged Sites for LPP Occipital and LPP Parietal Amplitudes*

| DV | Predictor | *β* | *SE* | *z* | *P>\|z\|* | *95% CI* | | *df* | *χ^2^* | *P> χ^2^* |
| --- | --- | --- | --- | --- | --- | --- | --- | --- | --- | --- |
| LPP LH Occipital |  |  |  |  |  |  |  | 6 | 55.26 | **<.001** |
|  | Age | -1.22 | 0.32 | 3.76 | **<.001** | -1.85 | -0.58 |  |  |  |
|  | Language | 0.02 | 0.04 | 0.55 | .584 | -0.05 | 0.10 |  |  |  |
|  | Emotion |  |  |  |  |  |  | 3 | 28.16 | **<.001** |
|  | Anger | 0.87 | 0.26 | 3.34 | **.002** | 0.36 | 1.37 |  |  |  |
|  | Happiness | 0.57 | 0.26 | 2.19 | **.029** | 0.06 | 1.08 |  |  |  |
|  | Fear | 1.34 | 0.26 | 5.17 | **<.001** | 0.83 | 1.85 |  |  |  |
|  | B.S |  |  |  |  |  |  | 1 | 13.79 | **<.001** |
|  | Female | -3.50 | 0.94 | -3.71 | **<.001** | -5.34 | -1.65 |  |  |  |
| LPP AV Occipital |  |  |  |  |  |  |  | 6 | 58.46 | **<.001** |
|  | Age | -1.36 | 0.29 | -4.65 | **<.001** | -1.93 | -0.79 |  |  |  |
|  | Language | 0.02 | 0.03 | 0.67 | .503 | -0.04 | 0.09 |  |  |  |
|  | Emotion |  |  |  |  |  |  | 3 | 22.77 | **<.001** |
|  | Anger | 0.69 | 0.24 | 2.86 | **.006** | 0.22 | 1.16 |  |  |  |
|  | Happiness | 0.58 | 0.24 | 2.40 | **.017** | 0.11 | 1.10 |  |  |  |
|  | Fear | 1.14 | 0.24 | 4.74 | **<.001** | 0.67 | 1.61 |  |  |  |
|  | B.S |  |  |  |  |  |  | 1 | 14.40 | **<.001** |
|  | Female | -3.22 | 0.85 | -3.79 | **<.001** | -4.88 | -1.56 |  |  |  |
| LPP LH Parietal |  |  |  |  |  |  |  | 6 | 37.35 | **<.001** |
|  | Age | -0.35 | 0.18 | -1.97 | **.048** | -0.69 | -0.002 |  |  |  |
|  | Language | -0.02 | 0.02 | -0.90 | .367 | -0.06 | 0.02 |  |  |  |
|  | Emotion |  |  |  |  |  |  | 3 | 27.12 | **<.001** |
|  | Anger | 0.55 | 0.18 | 3.06 | **.003** | 0.20 | 0.91 |  |  |  |
|  | Happiness | 0.46 | 0.18 | 2.52 | **.012** | 0.10 | 0.81 |  |  |  |
|  | Fear | 0.94 | 0.18 | 5.17 | **<.001** | 0.58 | 1.29 |  |  |  |
|  | B.S |  |  |  |  |  |  | 1 | 2.18 | .140 |
|  | Female | -0.75 | 0.51 | -1.48 | .140 | -1.75 | 0.25 |  |  |  |
| LPP AV Parietal |  |  |  |  |  |  |  | 6 | 48.92 | **<.001** |
|  | Age | -0.47 | 0.15 | -3.17 | **.002** | -0.76 | -0.18 |  |  |  |
|  | Language | -0.01 | 0.02 | -0.36 | .717 | -0.04 | 0.03 |  |  |  |
|  | Emotion |  |  |  |  |  |  | 3 | 30.24 | **<.001** |
|  | Anger | 0.58 | 0.19 | 3.30 | **.001** | 0.24 | 0.93 |  |  |  |
|  | Happiness | 0.61 | 0.18 | 3.45 | **.001** | 0.26 | 0.95 |  |  |  |
|  | Fear | 0.95 | 0.18 | 5.42 | **<.001** | 0.61 | 1.30 |  |  |  |
|  | B.S |  |  |  |  |  |  | 1 | 5.18 | **.023** |
|  | Female | -0.98 | 1.37 | -2.28 | **.023** | -1.82 | -0.14 |  |  |  |

*Note.* Significant main effects highlighted in bold. LH = left hemisphere; RH = right hemisphere; AV = averaged across hemispheres; Age = child’s age in years; B.S = Biological sex. Raw scores used for Language. FDR correction applied where appropriate. Emotion compared to Neutrality. Biological sex compared to male

**Table S2**

*Summary of Interaction Effects for Left Hemisphere and Averaged Sites for LPP Occipital and LPP Parietal Amplitudes*

| DV | Predictor | *β* | SE | z | P>\|z\| | *95% CI* | | *df* | *χ^2^* | *P> χ^2^* |
| --- | --- | --- | --- | --- | --- | --- | --- | --- | --- | --- |
| LPP LH Occipital |  |  |  |  |  |  |  | 13 | 64.32 | **<.001** |
|  | Age*Language | -0.02 | 0.02 | -1.26 | .206 | -0.06 | 0.01 |  |  |  |
|  | Age*Emotion |  |  |  |  |  |  | 3 | 2.93 | .402 |
|  | Anger | -0.10 | 0.18 | -0.58 | .561 | -0.45 | 0.24 |  |  |  |
|  | Happiness | 0.15 | 0.18 | 0.84 | .561 | -0.20 | 0.50 |  |  |  |
|  | Fear | -0.12 | 0.18 | -0.69 | .561 | -0.47 | 0.23 |  |  |  |
|  | Emotion*Language |  |  |  |  |  |  | 3 | 4.27 | .234 |
|  | Anger | -0.02 | 0.02 | -0.88 | .565 | -0.06 | 0.02 |  |  |  |
|  | Happiness | -0.04 | 0.02 | -2.01 | .135 | -0.08 | -0.001 |  |  |  |
|  | Fear | -0.01 | 0.02 | -0.58 | .565 | -0.05 | 0.03 |  |  |  |
| LPP AV Occipital |  |  |  |  |  |  |  | 13 | 68.81 | **<.001** |
|  | Age*Language | -0.02 | 0.02 | -1.08 | .280 | -2.26 | 1.63 |  |  |  |
|  | Age*Emotion |  |  |  |  |  |  | 3 | 1.66 | .647 |
|  | Anger | -0.11 | 0.16 | -0.69 | .697 | -0.44 | 0.21 |  |  |  |
|  | Happiness | 0.09 | 0.16 | 0.52 | .697 | -0.24 | 0.41 |  |  |  |
|  | Fear | -0.06 | 0.16 | -0.39 | .697 | -0.39 | 0.26 |  |  |  |
|  | Emotion*Language |  |  |  |  |  |  | 3 | 6.10 | .107 |
|  | Anger | -0.02 | 0.02 | -1.16 | .369 | -0.06 | 0.02 |  |  |  |
|  | Happiness | -0.04 | 0.02 | -2.26 | .072 | -0.08 | -0.01 |  |  |  |
|  | Fear | -0.01 | 0.02 | -0.33 | .745 | -0.04 | 0.03 |  |  |  |
| LPP LH Parietal |  |  |  |  |  |  |  | 13 | 43.43 | **<.001** |
|  | Age*Language | -0.01 | 0.01 | -1.33 | .182 | -0.03 | 0.01 |  |  |  |
|  | Age*Emotion |  |  |  |  |  |  | 3 | 2.99 | .394 |
|  | Anger | -0.02 | 0.12 | -0.20 | .844 | -0.27 | 0.22 |  |  |  |
|  | Happiness | 0.14 | 0.12 | 1.10 | .816 | -0.11 | 0.38 |  |  |  |
|  | Fear | -0.07 | 0.12 | -0.53 | .844 | -0.31 | 0.18 |  |  |  |
|  | Emotion*Language |  |  |  |  |  |  | 3 | 1.17 | .760 |
|  | Anger | -0.003 | 0.01 | -0.23 | .817 | -0.03 | 0.03 |  |  |  |
|  | Happiness | -0.02 | 0.01 | -1.03 | .817 | -0.04 | 0.01 |  |  |  |
|  | Fear | -0.01 | 0.01 | -0.52 | .817 | -0.04 | 0.02 |  |  |  |
| LPP AV Parietal |  |  |  |  |  |  |  | 13 | 56.04 | **<.001** |
|  | Age*Language | 0.0003 | 0.01 | 0.04 | .971 | -0.02 | 0.02 |  |  |  |
|  | Age*Emotion |  |  |  |  |  |  | 3 | 0.92 | .821 |
|  | Anger | -0.03 | 0.12 | -0.22 | .918 | -0.26 | 0.21 |  |  |  |
|  | Happiness | 0.08 | 0.12 | 0.70 | .918 | -0.15 | 0.32 |  |  |  |
|  | Fear | 0.01 | 0.12 | 0.10 | .918 | -0.22 | 0.25 |  |  |  |
|  | Emotion*Language |  |  |  |  |  |  | 3 | 5.83 | .120 |
|  | Anger | -0.01 | 0.01 | -0.86 | .585 | -0.04 | 0.02 |  |  |  |
|  | Happiness | -0.03 | 0.01 | -2.27 | .072 | -0.06 | -0.004 |  |  |  |
|  | Fear | -0.01 | 0.01 | -0.41 | .682 | -0.03 | 0.02 |  |  |  |

*Note*. Significant interaction effects highlighted in bold. LH = left hemisphere; AV = averaged across hemispheres; Age = child’s age in years; Raw scores used for Language. FDR correction applied where appropriate.

**Table S3**

*Summary of LPP Main Effects Averaged Across Occipital and Parietal sites*

| DV | Predictor | *β* | SE | z | P>\|z\| | 95% CI | | df | *χ*^2^ | P> *χ*^2^ |
| --- | --- | --- | --- | --- | --- | --- | --- | --- | --- | --- |
| LPP RH |  |  |  |  |  |  |  | 6 | 55.01 | **<.001** |
|  | Age | -1.08 | 0.21 | -5.03 | **<.001** | -1.50 | -0.66 |  |  |  |
|  | Language | 0.01 | 0.03 | 0.59 | .558 | -0.03 | 0.06 |  |  |  |
|  | Emotion |  |  |  |  |  |  | 3 | 19.90 | **<.001** |
|  | Anger | 0.55 | 0.22 | 2.47 | **.014** | 0.11 | 0.99 |  |  |  |
|  | Happiness | 0.62 | 0.22 | 2.79 | **.008** | 0.18 | 1.06 |  |  |  |
|  | Fear | 0.98 | 0.22 | 4.41 | **<.001** | 0.55 | 1.42 |  |  |  |
|  | B.S |  |  |  |  |  |  | 1 | 7.99 | **.005** |
|  | Female | -1.76 | 0.62 | -2.83 | **.005** | -2.98 | -0.54 |  |  |  |
| LPP LH |  |  |  |  |  |  |  | 6 | 53.94 | **<.001** |
|  | Age | -0.78 | 0.23 | -3.42 | **.001** | -1.23 | -0.33 |  |  |  |
|  | Language | 0.001 | 0.03 | 0.04 | .966 | -0.05 | 0.05 |  |  |  |
|  | Emotion |  |  |  |  |  |  | 3 | 30.81 | **<.001** |
|  | Anger | 0.71 | 0.21 | 3.40 | **.002** | 0.30 | 1.12 |  |  |  |
|  | Happiness | 0.51 | 0.21 | 2.47 | **.014** | 0.11 | 0.92 |  |  |  |
|  | Fear | 1.14 | 0.21 | 5.46 | **<.001** | 0.73 | 1.55 |  |  |  |
|  | B.S |  |  |  |  |  |  | 1 | 10.20 | **.001** |
|  | Female | -2.13 | 0.67 | -3.19 | **.001** | -3.43 | -0.82 |  |  |  |
| LPP AV |  |  |  |  |  |  |  | 6 | 61.17 | **<.001** |
|  | Age | -0.91 | 0.20 | -4.49 | **<.001** | -1.31 | -0.51 |  |  |  |
|  | Language | 0.01 | 0.02 | 0.35 | .727 | -0.04 | 0.05 |  |  |  |
|  | Emotion |  |  |  |  |  |  | 3 | 27.25 | **<.001** |
|  | Anger | 0.64 | 0.20 | 3.14 | **.003** | 0.24 | 1.03 |  |  |  |
|  | Happiness | 0.60 | 0.20 | 2.94 | **.003** | 0.20 | 0.99 |  |  |  |
|  | Fear | 1.05 | 0.20 | 5.18 | **<.001** | 0.65 | 1.45 |  |  |  |
|  | B.S |  |  |  |  |  |  | 1 | 12.59 | **<.001** |
|  | Female | -2.10 | 0.59 | -3.55 | **<.001** | -3.26 | -0.94 |  |  |  |

*Note.* Significant main effects highlighted in bold. RH = right hemisphere; LH = left hemisphere; AV – averaged across hemispheres; Age = child’s age in years; B.S = Biological sex. Raw scores used for Language. FDR correction applied where appropriate. Emotion compared to Neutrality. Biological sex compared to male

**Table S4**

*Summary of LPP Interaction Effects Averaged Across Occipital and Parietal sites*

| DV | Predictor | *β* | SE | z | P>\|z\| | 95% CI | | df | *χ*^2^ | P> *χ*^2^ |
| --- | --- | --- | --- | --- | --- | --- | --- | --- | --- | --- |
| LPP RH |  |  |  |  |  |  |  | 13 | 67.85 | **<.001** |
|  | Age*Language | 0.003 | 0.01 | 0.22 | .827 | -2.65 | 0.23 |  |  |  |
|  | Age*Emotion |  |  |  |  |  |  | 3 | 0.98 | .807 |
|  | Anger | -0.11 | 0.15 | -0.72 | .995 | -0.41 | 0.19 |  |  |  |
|  | Happiness | 0.03 | 0.15 | 0.19 | .995 | -0.27 | 0.32 |  |  |  |
|  | Fear | 0.001 | 0.15 | 0.01 | .995 | -0.30 | 0.30 |  |  |  |
|  | Emotion*Language |  |  |  |  |  |  | 3 | 8.26 | **.041** |
|  | Anger | -0.02 | 0.02 | -1.17 | .365 | -0.06 | 0.01 |  |  |  |
|  | Happiness | -0.04 | 0.02 | -2.43 | **.045** | -0.08 | -0.01 |  |  |  |
|  | Fear | 0.001 | 0.02 | 0.06 | .955 | -0.03 | 0.04 |  |  |  |
| LPP LH |  |  |  |  |  |  |  | 13 | 62.18 | **<.001** |
|  | Age*Language | -0.02 | 0.01 | -1.41 | .160 | -0.04 | 0.01 |  |  |  |
|  | Age*Emotion |  |  |  |  |  |  | 3 | 3.25 | .355 |
|  | Anger | -0.06 | 0.14 | -0.45 | .655 | -0.34 | 0.22 |  |  |  |
|  | Happiness | 0.14 | 0.14 | 1.00 | .655 | -0.14 | 0.42 |  |  |  |
|  | Fear | -0.09 | 0.14 | -0.66 | .655 | -0.37 | 0.19 |  |  |  |
|  | Emotion*Language |  |  |  |  |  |  | 3 | 2.97 | .400 |
|  | Anger | -0.01 | 0.02 | -0.65 | .561 | -0.04 | 0.02 |  |  |  |
|  | Happiness | -0.03 | 0.02 | -1.69 | .273 | -0.06 | 0.0045 |  |  |  |
|  | Fear | -0.01 | 0.02 | -0.58 | .561 | -0.04 | 0.02 |  |  |  |
| LPP AV |  |  |  |  |  |  |  | 13 | 70.33 | **<.001** |
|  | Age*Language | -0.01 | 0.01 | -0.76 | .448 | -0.03 | 0.01 |  |  |  |
|  | Age*Emotion |  |  |  |  |  |  | 3 | 1.34 | .720 |
|  | Anger | -0.07 | 0.14 | -0.51 | .852 | -0.34 | 0.20 |  |  |  |
|  | Happiness | 0.09 | 0.14 | 0.62 | .852 | -0.19 | 0.36 |  |  |  |
|  | Fear | -0.03 | 0.14 | -0.19 | .852 | -0.30 | 0.24 |  |  |  |
|  | Emotion*Language |  |  |  |  |  |  | 3 | 6.31 | .098 |
|  | Anger | -0.02 | 0.02 | -1.06 | .432 | -0.05 | 0.01 |  |  |  |
|  | Happiness | -0.04 | 0.02 | -2.33 | .060 | -0.07 | -0.01 |  |  |  |
|  | Fear | -0.01 | 0.02 | -0.37 | .711 | -0.04 | 0.03 |  |  |  |

*Note*. Significant interaction effects highlighted in bold. RH = right hemisphere; LH = left hemisphere; AV – averaged across hemispheres; Age = child’s age in years; Raw scores used for Language. FDR correction applied where appropriate.

**Assumption testing**

Inspection of the data revealed no missing data. Data were screened for univariate outliers, with kurtosis and skewness values suggesting no extreme cases. Multivariate outliers were screened, with Mahalanobis values showing that no cases exceeded the critical value of 0.001 with 4 degrees of freedom (18.467). Consistent with Hoaglin and Welsch (1978), Cooks distance and leverage values further indicated no multivariate outliers. Linearity, normality, multicollinearity, homoscedasticity, and homogeneity assumptions were met.

**Data analysis script**

mixed P1_Amp c.RS c.Age i.EmoNum i.GenNum, || ID:

contrast i.EmoNum i.GenNum

mixed P1_Amp c.RS c.Age c.RS#c.Age i.EmoNum i.GenNum c.Age#i.EmoNum c.RS#i.EmoNum, || ID:

contrast i.EmoNum i.GenNum c.Age#i.EmoNum c.RS#i.EmoNum

mixed P1_Lat c.RS c.Age i.EmoNum i.GenNum, || ID:

contrast i.EmoNum i.GenNum

mixed P1_Lat c.RS c.Age c.RS#c.Age i.EmoNum i.GenNum c.Age#i.EmoNum c.RS#i.EmoNum, || ID:

contrast i.EmoNum i.GenNum c.Age#i.EmoNum c.RS#i.EmoNum

mixed N170_Amp c.RS c.Age i.EmoNum i.GenNum, || ID:

contrast i.EmoNum i.GenNum

mixed N170_Amp c.RS c.Age c.RS#c.Age i.EmoNum i.GenNum c.Age#i.EmoNum c.RS#i.EmoNum, || ID:

contrast i.EmoNum i.GenNum c.Age#i.EmoNum c.RS#i.EmoNum

mixed N170_Lat c.RS c.Age i.EmoNum i.GenNum, || ID:

contrast i.EmoNum i.GenNum

mixed N170_Lat c.RS c.Age c.RS#c.Age i.EmoNum i.GenNum c.Age#i.EmoNum c.RS#i.EmoNum, || ID:

contrast i.EmoNum i.GenNum c.Age#i.EmoNum c.RS#i.EmoNum

mixed LPPPar_Amp c.RS c.Age i.EmoNum i.GenNum, || ID:

contrast i.EmoNum i.GenNum

mixed LPPPar_Amp c.RS c.Age c.RS#c.Age i.EmoNum i.GenNum c.Age#i.EmoNum c.RS#i.EmoNum, || ID:

contrast i.EmoNum i.GenNum c.Age#i.EmoNum c.RS#i.EmoNum

mixed LPPOcc_Amp c.RS c.Age i.EmoNum i.GenNum, || ID:

contrast i.EmoNum i.GenNum

mixed LPPOcc_Amp c.RS c.Age c.RS#c.Age i.EmoNum i.GenNum c.Age#i.EmoNum c.RS#i.EmoNum, || ID:

contrast i.EmoNum i.GenNum c.Age#i.EmoNum c.RS#i.EmoNum

**Table S5**

*Summary of Main Effects for Sample With Youngest Participants Removed*

| DV | Predictor | *β* | SE | z | P>\|z\| | 95% CI | | df | *χ*^2^ | P> *χ*^2^ |
| --- | --- | --- | --- | --- | --- | --- | --- | --- | --- | --- |
| P100 Amplitude |  |  |  |  |  |  |  | 6 | 19.53 | **.003** |
|  | Age | -2.09 | 0.76 | -2.75 | **.006** | -3.58 | -0.60 |  |  |  |
|  | Language | 0.01 | 0.08 | 0.18 | .856 | -0.14 | 0.17 |  |  |  |
|  | Emotion |  |  |  |  |  |  | 3 | 5.15 | .161 |
|  | Anger | 0.55 | 0.34 | 1.64 | .101 | -0.11 | 1.21 |  |  |  |
|  | Happiness | 0.65 | 0.34 | 1.93 | .081 | -0.01 | 1.31 |  |  |  |
|  | Fear | 0.65 | 0.34 | 1.93 | .081 | -0.01 | 1.31 |  |  |  |
|  | B.S |  |  |  |  |  |  | 1 | 7.70 | **.006** |
|  | Female | -5.58 | 2.01 | -2.78 | **.006** | -9.52 | -1.64 |  |  |  |
| P100 Latency |  |  |  |  |  |  |  | 6 | 11.32 | .079 |
|  | Age | -1.49 | 1.02 | -1.47 | .142 | -3.49 | 0.50 |  |  |  |
|  | Language | 0.22 | 0.11 | 2.00 | **.046** | 0.004 | 0.43 |  |  |  |
|  | Emotion |  |  |  |  |  |  | 3 | 2.45 | .485 |
|  | Anger | -1.21 | 1.04 | -1.17 | .363 | -3.25 | 0.82 |  |  |  |
|  | Happiness | -1.42 | 1.04 | -1.37 | .363 | -3.45 | 0.61 |  |  |  |
|  | Fear | -0.45 | 1.04 | -0.43 | .665 | -2.48 | 1.58 |  |  |  |
|  | B.S |  |  |  |  |  |  | 1 | 5.20 | **.023** |
|  | Female | -6.13 | 2.69 | -2.28 | **.023** | -11.40 | -0.86 |  |  |  |
| N170 Amplitude |  |  |  |  |  |  |  | 6 | 12.75 | **.047** |
|  | Age | 0.83 | 0.32 | 2.60 | **.009** | 0.20 | 1.46 |  |  |  |
|  | Language | 0.01 | 0.03 | 0.19 | .853 | -0.06 | 0.07 |  |  |  |
|  | Emotion |  |  |  |  |  |  | 3 | 4.58 | .205 |
|  | Anger | -0.44 | 0.27 | -1.65 | .297 | -0.97 | 0.08 |  |  |  |
|  | Happiness | -0.23 | 0.27 | -0.86 | .581 | -0.76 | 0.29 |  |  |  |
|  | Fear | 0.07 | 0.27 | 0.27 | .784 | -0.45 | 0.60 |  |  |  |
|  | B.S |  |  |  |  |  |  | 1 | 0.44 | .505 |
|  | Female | 0.56 | 0.85 | 0.67 | .505 | -1.10 | 2.22 |  |  |  |
| N170 Latency |  |  |  |  |  |  |  | 6 | 17.85 | **.007** |
|  | Age | -4.14 | 1.21 | -3.44 | **.001** | -6.49 | -1.78 |  |  |  |
|  | Language | 0.22 | 0.13 | 1.75 | .080 | -0.03 | 0.48 |  |  |  |
|  | Emotion |  |  |  |  |  |  | 3 | 3.72 | .294 |
|  | Anger | -1.81 | 1.85 | -0.98 | .328 | -5.44 | 1.82 |  |  |  |
|  | Happiness | -2.30 | 1.85 | -1.24 | .323 | -5.93 | 1.33 |  |  |  |
|  | Fear | -3.51 | 1.85 | -1.90 | .174 | -7.14 | 0.12 |  |  |  |
|  | B.S |  |  |  |  |  |  | 1 | 3.35 | **.**067 |
|  | Female | -5.82 | 3.18 | -1.83 | .067 | -12.05 | 0.41 |  |  |  |
| LPP Occipital Amplitude |  |  |  |  |  |  |  | 6 | 44.54 | **<.001** |
|  | Age | -1.65 | 0.35 | -4.78 | **<.001** | -2.33 | -0.97 |  |  |  |
|  | Language | 0.03 | 0.04 | 0.92 | .359 | -0.04 | 0.11 |  |  |  |
|  | Emotion |  |  |  |  |  |  | 3 | 17.66 | **.001** |
|  | Anger | 0.48 | 0.27 | 1.82 | .070 | -0.04 | 1.01 |  |  |  |
|  | Happiness | 0.59 | 0.27 | 2.22 | **.041** | 0.07 | 1.11 |  |  |  |
|  | Fear | 1.12 | 0.27 | 4.18 | **<.001** | 0.59 | 1.64 |  |  |  |
|  | B.S |  |  |  |  |  |  | 1 | 5.86 | **.016** |
|  | Female | -2.21 | 0.91 | -2.42 | **.015** | -4.00 | -0.42 |  |  |  |
| LPP Parietal Amplitude |  |  |  |  |  |  |  | 6 | 37.46 | **<.001** |
|  | Age | -0.49 | 0.18 | -2.69 | **.007** | -0.85 | -0.13 |  |  |  |
|  | Language | 0.01 | 0.02 | 0.57 | .566 | -0.03 | 0.05 |  |  |  |
|  | Emotion |  |  |  |  |  |  | 3 | 23.72 | **<.001** |
|  | Anger | 0.50 | 0.20 | 2.56 | .**010** | 0.12 | 0.87 |  |  |  |
|  | Happiness | 0.56 | 0.20 | 2.90 | **.006** | 0.18 | 0.94 |  |  |  |
|  | Fear | 0.94 | 0.20 | 4.84 | **<.001** | 0.56 | 1.32 |  |  |  |
|  | B.S |  |  |  |  |  |  | 1 | 7.98 | **.005** |
|  | Female | -1.36 | 0.48 | -2.83 | **.005** | -2.30 | -0.42 |  |  |  |

*Note.* Significant main effects highlighted in bold. Age = child’s age in years; B.S = Biological sex. Raw scores used for Language. FDR correction applied where appropriate. Emotion compared to Neutrality. Biological sex compared to male

**Table S6**

*Summary of Interaction Effects for Sample With Youngest Participants Removed*

| DV | Predictor | *β* | SE | z | P>\|z\| | 95% CI | | df | *χ*^2^ | P> *χ*^2^ |
| --- | --- | --- | --- | --- | --- | --- | --- | --- | --- | --- |
| P100 Amplitude |  |  |  |  |  |  | |  |  |  |
|  | Age*Language | 0.001 | 0.05 | 0.02 | .987 | -0.10 | 0.11 |  |  |  |
|  | Age*Emotion |  |  |  |  |  |  | 3 | 1.48 | .688 |
|  | Anger | -0.22 | 0.25 | -0.87 | .813 | -0.72 | 0.28 |  |  |  |
|  | Happiness | 0.05 | 0.25 | 0.18 | .856 | -0.45 | 0.54 |  |  |  |
|  | Fear | -0.15 | 0.25 | -0.61 | .813 | -0.65 | 0.34 |  |  |  |
|  | Emotion*Language |  |  |  |  |  |  | 3 | 2.04 | .563 |
|  | Anger | 0.01 | 0.03 | 0.32 | .760 | -0.04 | 0.06 |  |  |  |
|  | Happiness | -0.01 | 0.03 | -0.31 | .760 | -0.06 | 0.04 |  |  |  |
|  | Fear | 0.03 | 0.03 | 1.05 | .760 | -0.02 | 0.08 |  |  |  |
| P100 Latency |  |  |  |  |  |  |  |  |  |  |
|  | Age*Language | -0.03 | 0.07 | -0.41 | .681 | -0.17 | 0.11 |  |  |  |
|  | Age*Emotion |  |  |  |  |  |  | 3 | 10.19 | **.017** |
|  | Anger | -2.27 | 0.77 | -2.94 | **.009** | -3.78 | -0.76 |  |  |  |
|  | Happiness | -0.39 | 0.77 | -0.51 | .612 | -1.90 | 1.12 |  |  |  |
|  | Fear | -1.22 | 0.77 | -1.59 | .168 | -2.73 | 0.29 |  |  |  |
|  | Emotion*Language |  |  |  |  |  |  | 3 | 9.20 | **.027** |
|  | Anger | 0.24 | 0.08 | 2.91 | **.012** | 0.08 | 0.40 |  |  |  |
|  | Happiness | 0.14 | 0.08 | 1.68 | .093 | -0.02 | 0.30 |  |  |  |
|  | Fear | 0.18 | 0.08 | 2.20 | **.042** | 0.02 | 0.34 |  |  |  |
| N170 Amplitude |  |  |  |  |  |  |  |  |  |  |
|  | Age*Language | 0.01 | 0.02 | 0.45 | .651 | -0.03 | 0.05 |  |  |  |
|  | Age*Emotion |  |  |  |  |  |  | 3 | 9.64 | **.022** |
|  | Anger | -0.15 | 0.20 | -0.75 | .991 | -0.54 | 0.24 |  |  |  |
|  | Happiness | 0.25 | 0.20 | 1.23 | .327 | -0.15 | 0.64 |  |  |  |
|  | Fear | -0.36 | 0.20 | -1.78 | .228 | -0.75 | 0.04 |  |  |  |
|  | Emotion*Language |  |  |  |  |  |  | 3 | 2.47 | .481 |
|  | Anger | -0.0002 | 0.02 | -0.01 | .991 | -0.04 | 0.04 |  |  |  |
|  | Happiness | -0.02 | 0.02 | -0.84 | .696 | -0.06 | 0.02 |  |  |  |
|  | Fear | 0.02 | 0.02 | 0.73 | .606 | -0.03 | 0.06 |  |  |  |
| N170 Latency |  |  |  |  |  |  |  |  |  |  |
|  | Age*Language | 0.11 | 0.08 | 1.29 | .197 | -0.06 | 0.27 |  |  |  |
|  | Age*Emotion |  |  |  |  |  |  | 3 | 4.37 | .224 |
|  | Anger | -2.33 | 1.39 | -1.67 | .136 | -5.06 | 0.40 |  |  |  |
|  | Happiness | -2.08 | 1.39 | -1.49 | .136 | -4.81 | 0.65 |  |  |  |
|  | Fear | -2.60 | 1.39 | -1.87 | .136 | -5.33 | 0.12 |  |  |  |
|  | Emotion*Language |  |  |  |  |  |  | 3 | 1.51 | .680 |
|  | Anger | 0.04 | 0.15 | 0.30 | .782 | -0.25 | 0.34 |  |  |  |
|  | Happiness | -0.04 | 0.15 | -0.28 | .782 | -0.33 | 0.25 |  |  |  |
|  | Fear | -0.13 | 0.15 | -0.88 | .782 | -0.42 | 0.16 |  |  |  |
| LPP Occipital Amplitude |  |  |  |  |  |  |  |  |  |  |
|  | Age*Language | -0.01 | 0.02 | -0.53 | .594 | -0.06 | 0.03 |  |  |  |
|  | Age*Emotion |  |  |  |  |  |  | 3 | 1.38 | .711 |
|  | Anger | -0.17 | 0.20 | -0.84 | .603 | -0.56 | 0.22 |  |  |  |
|  | Happiness | -0.01 | 0.20 | -0.07 | .943 | -0.41 | 0.38 |  |  |  |
|  | Fear | -0.18 | 0.20 | -0.89 | .603 | -0.57 | 0.21 |  |  |  |
|  | Emotion*Language |  |  |  |  |  |  | 3 | 8.67 | **.034** |
|  | Anger | -0.02 | 0.02 | -1.11 | .399 | -0.07 | 0.02 |  |  |  |
|  | Happiness | -0.05 | 0.02 | -2.38 | **.051** | -0.09 | -0.01 |  |  |  |
|  | Fear | 0.01 | 0.02 | 0.25 | .799 | -0.04 | 0.05 |  |  |  |
| LPP Parietal Amplitude |  |  |  |  |  |  |  |  |  |  |
|  | Age*Language | 0.01 | 0.01 | 0.56 | .574 | -0.02 | 0.03 |  |  |  |
|  | Age*Emotion |  |  |  |  |  |  | 3 | 0.36 | .949 |
|  | Anger | -0.06 | 0.14 | -0.41 | .950 | -0.34 | 0.22 |  |  |  |
|  | Happiness | 0.02 | 0.14 | 0.17 | .950 | -0.26 | 0.31 |  |  |  |
|  | Fear | -0.01 | 0.15 | -0.06 | .950 | -0.29 | 0.27 |  |  |  |
|  | Emotion*Language |  |  |  |  |  |  | 3 | 9.13 | **.028** |
|  | Anger | -0.01 | 0.02 | -0.94 | .521 | -0.04 | 0.02 |  |  |  |
|  | Happiness | -0.04 | 0.02 | -2.50 | **.039** | -0.07 | -0.01 |  |  |  |
|  | Fear | 0.003 | 0.02 | 0.21 | .831 | -0.03 | 0.03 |  |  |  |

*Note.* Significant main effects highlighted in bold. Age = child’s age in years; B.S = Biological sex. Raw scores used for Language. FDR correction applied where appropriate.
